# Facilitators and barriers to engaging patient partners in knowledge syntheses: A stage-based approach

**DOI:** 10.1017/rsm.2026.10099

**Published:** 2026-06-15

**Authors:** Anna Maria Chudyk, Sasha M. Kullman, Jenna Villarba, Caroline Monnin, Janet Gunderson, Jenna Kedy, Tracy Slaney, Andrea C. Tricco

**Affiliations:** 1 College of Pharmacy, University of Manitoba, Canada; 2 Neil John Maclean Health Sciences Library, University of Manitoba, Canada; 3 Person with Lived Experience, College of Pharmacy, University of Manitoba, Canada; 4 Knowledge Translation Program, Li Ka Shing Knowledge Institute, St. Michael’s Hospital, Unity Health Toronto, Canada; 5 Dalla Lana School of Public Health, Epidemiology Division, University of Toronto, Canada

**Keywords:** knowledge synthesis, lived experience in health research, participatory research, patient engagement, patient and public involvement

## Abstract

Knowledge synthesis involves bringing together findings from individual research studies to answer a specific question or explore a particular topic, often at a global level. People with lived or living experience of health conditions or of accessing healthcare services (PWLE), such as patients and their families or friends, can provide valuable perspectives throughout this process. PWLE involvement can help shape the design and conduct of a knowledge synthesis to better reflect real-world concerns through a process called patient engagement. Patient engagement is the meaningful and active involvement of PWLE throughout the research process. While previous research shows that PWLE can contribute meaningfully when appropriate supports are in place, there is limited practical guidance on how to enact patient engagement in knowledge syntheses. This tutorial paper aims to guide researchers and PWLE who would like to engage in knowledge syntheses together. Building on existing research, our team (comprised of PWLE, researchers, and an academic librarian) shares lessons we have learned from working together throughout the stages of a knowledge synthesis. We present considerations for successful patient engagement when research teams are: (1) planning to engage, (2) recruiting PWLE, (3) establishing roles and rapport, (4) supporting capacity development, (5) conducting the knowledge synthesis, and (6) mobilizing findings. We also identify 12 common barriers to patient engagement and offer strategies to address them, alongside recommendations for integrating PWLE across every stage of the knowledge synthesis process.

## Highlights

### What is already known?

- People with lived or living experience of health conditions or accessing healthcare services (PWLE), such as patients and their families or friends, can offer valuable insights when planning and conducting knowledge syntheses in health research.

### What is new?

- There is limited practical guidance on how to meaningfully engage PWLE in knowledge syntheses. Drawing on our team’s lived, living, and learned experiences, we provide hands-on strategies for supporting PWLE engagement across every stage of a knowledge synthesis.

### Potential impact for RSM readers

- This tutorial paper offers concrete, experience-based recommendations and tools that researchers and PWLE can use to collaborate more effectively throughout knowledge syntheses, helping to improve the relevance of synthesized evidence to PWLE’s needs and priorities.

## 1 Background

Whether you are making a policy decision, creating clinical guidelines, or navigating your own health journey, having a clear picture of the evidence matters. Knowledge synthesis, also known as evidence synthesis, is a way of bringing together and making sense of findings from individual research studies that focus on a specific question or topic, often on a global scale.^1^ Over time, this field has grown and taken on many forms, such as systematic reviews, meta-analyses, rapid reviews, scoping reviews, and umbrella reviews (also called overviews of reviews).^1^^,^ ^2^ While each type of knowledge synthesis uses slightly different methods, they all follow a structured and transparent process that helps ensure the results are reliable and accurately reflect the combined evidence.^3^^,^ ^4^ These syntheses are valuable for shaping future research and are key tools for sharing knowledge. They help connect research with real-world decision-making by informing policy briefs, creating tools to support patient decisions, developing clinical guidelines, or improving how health services are planned and delivered.^1^^,^ ^2^^,^ ^5^

As these methods have continued to evolve, so too has the recognition that bringing a wide range of perspectives into the process is essential.^6^^,^ ^7^ People with lived or living experience of health conditions and/or accessing healthcare services (PWLE)—such as patients and their care partners (e.g., family and friends who support their care)—can offer important insights when planning and conducting research, including knowledge syntheses.^8^ There is a growing global movement, often called patient engagement, public involvement, or community and consumer involvement, that focuses on ensuring PWLE are meaningfully and actively involved in research through shared decision-making in the research process.^9^^–^ ^11^

Within this movement, PWLE input is applied in tandem with research evidence, theory, and clinical or professional expertise to guide research planning, conduct, decision-making, and outputs.^12^ This process can help ensure that research findings are relevant to the individuals and groups they impact.^13^^,^ ^14^ While recognizing the value of PWLE engagement to gain insight into lived experiences, it is also important to acknowledge that individual perspectives can vary widely. Therefore, while PWLE share valuable personal experiences that may resonate with others, they do not necessarily speak for entire groups or communities.^15^^,^ ^16^

Several studies have looked at how PWLE have been involved in knowledge syntheses, whether through shared reflections,^17^^–^ ^19^ calls for greater involvement,^7^^,^ ^17^ or papers focused on methods.^13^ What this work shows is that when the right supports are in place, and barriers are addressed, PWLE add meaningful insights to knowledge syntheses that are unique and complementary to the perspectives of other research team members.^13^^,^ ^17^ These contributions often stem from thoughtful discussions about what matters most to them and how they would like to be involved. Still, there is little practical guidance available on how to support meaningful PWLE engagement in these projects. That is where our article comes in. Building on the findings of Agyei-Manu et al.,^13^ our team (comprised of PWLE, researchers, and an academic librarian) offer our own lived, living, and learned experiences of engaging together across the stages of a knowledge synthesis. Our goal with this tutorial paper is to offer hands-on ideas for supporting engagement and tackling the challenges that can come up along the way.

## 2 Methods

We used the supports and challenges to patient engagement in knowledge syntheses identified by Agyei-Manu et al.,^13^ as a structural framework that guided our conversations and reflections when writing this article. To this framework, we add our experiences of collaborating as a group of academic researchers and PWLE to suggest practical strategies for facilitating engagement in knowledge syntheses and mitigating barriers.

### 2.1 Terminology

When patients and care partners join a research team, it is essential for the entire team to collectively determine the language used to describe those engaged and the nature of their engagement.^20^ In this work, we use the term PWLE to refer to members of our research team who represented the patient and care partner perspective. This terminology was chosen by PWLE because it acknowledges their diverse experiences of health, healthcare systems, and research engagement. However, we recognize that research partners in other contexts may prefer different terminology.

### 2.2 Team composition

Our team consisted of two academic researchers (AMC and ACT), two PhD Students (SK and JV), an academic librarian (CM), and four PWLE—three of whom chose to be listed as co-authors (JG, JK, and TS). This article is a secondary output of an ongoing scoping review of patient engagement in pharmacy practice research.^21^ The PWLE were actively involved in the scoping review and drew from this and other experiences to inform our present work. Our scoping review is currently at the data analysis stage.

Our PWLE collaborators were intentionally selected because they represented a diversity of perspectives. Specifically, they lived in four provinces (two in urban settings and two in rural), represented a mix of patient and care partner perspectives, had different levels of experience with knowledge syntheses and patient engagement (ranging from very experienced to just getting started) and represented a variety of age groups, including one youth PWLE. Beyond this project, all PWLE were actively involved in provincial or national patient and public advisory groups, bringing valuable experience in patient-oriented research.

Our team conducted this research at a Canadian academic institution, and our experiences and recommendations are framed within that context. Engaging PWLE in research is a rapidly growing practice in Canada and is supported by research funders, such as the Canadian Institutes of Health Research, through its Strategy for Patient-Oriented Research (SPOR).^9^ Canada is also home to many rich networks of patients, families, and PWLE who are engaged in shaping research and building capacity for engagement. Within this context, our institution and the colleagues with whom we collaborate are generally receptive to engaging PWLE in research, and our work is supported. We encourage readers to reflect on the similarities and differences in their individual research contexts as they engage with our recommendations, and to assess whether additional capacity building is needed before implementation.

### 2.3 Engagement in the present work

The PWLE who co-authored this article were engaged at the level of “collaborate,” meaning they were equal members of the research team and had an active role in decision-making.^22^ Specifically, they co-developed and refined this article through virtual meetings where they provided verbal input on each section. They also provided written feedback on each draft. PWLE roles and responsibilities were mutually established through the co-development of a terms of reference at the outset of our research (Supplementary File 1).^14^ Through this process, we ensured our engagement practices followed the core principles set out by the Canadian Institutes of Health Research Strategy for Patient-Oriented Research, our nation’s major funder and champion of patient engagement in research. These principles are mutual respect, inclusiveness, co-building, and support.^9^ PWLE were compensated at a rate of $25 per hour for their time spent on study-related activities, including training, meeting preparation, and document review or revision, in accordance with the agreed-upon terms.^23^

## 3 Results

**Figure 1:**
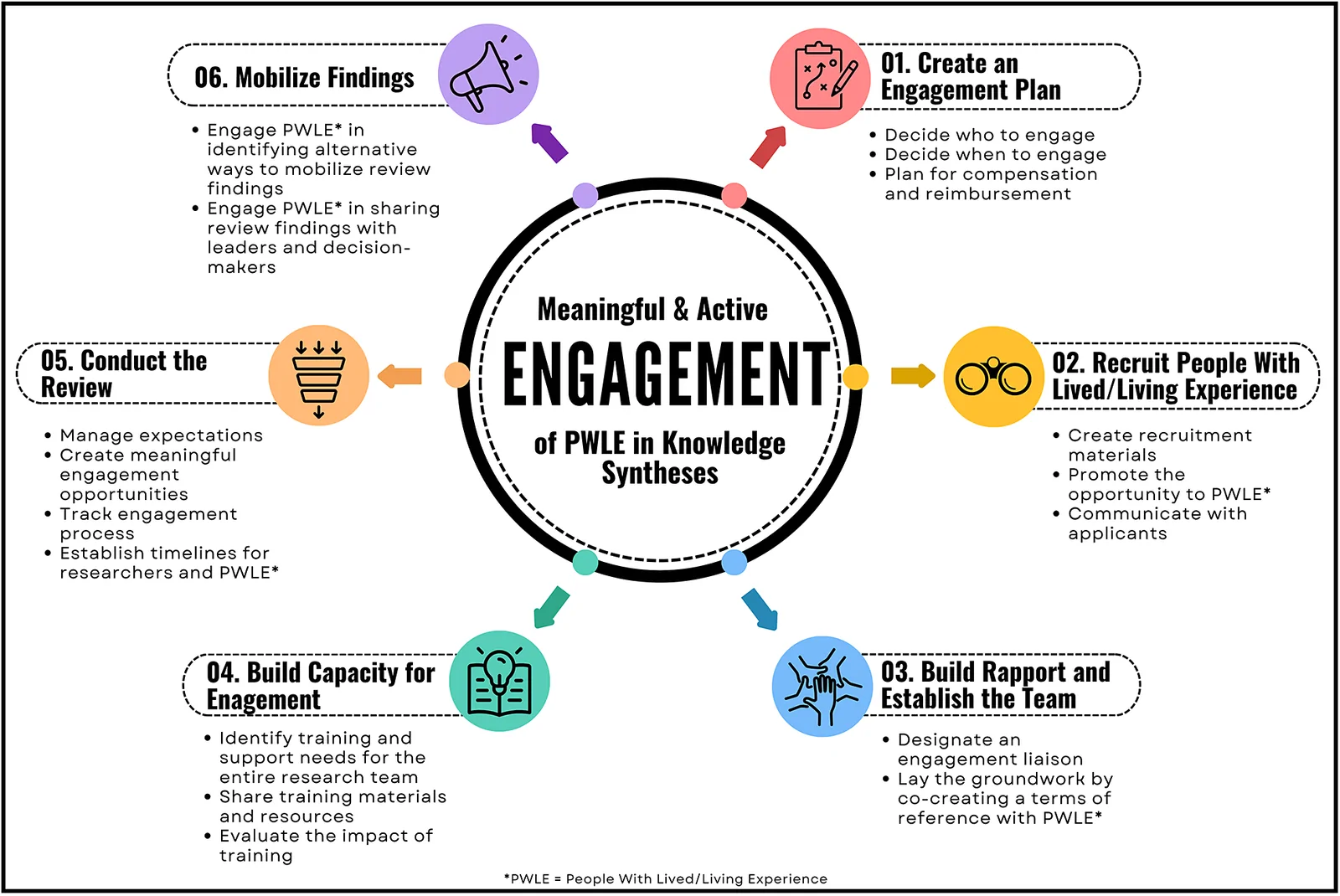
Overview of key considerations for meaningful and active engagement of PWLE across the stages of a knowledge synthesis. Figure 1 long description.

This tutorial paper outlines key considerations and strategies for engaging PWLE in knowledge syntheses across each stage of the research process (summarized in Figure 1). We hope researchers and PWLE can use this document as a practical guide in their work. Throughout the next sections, we encourage the reader to reflect on the following questions adapted from Richards et al.^24^ to help internalize key takeaways: *Have you seen these situations in your own work? Have you spoken to someone with lived/living experience about similar situations? What have you done or would you do to mitigate these challenges or avoid them in the future? Who has the power and privilege in this situation?*

## 4 Stage 1: Creating an engagement plan

Deciding to engage PWLE in a knowledge synthesis begins with a clear and thoughtful plan, which should be revised with PWLE once they join the study if the project is researcher-initiated or there is a change in PWLE membership over time. This plan should consider who will be engaged, when the opportune moments to engage are, and how PWLE will be compensated for the time and expertise they bring to the research.^13^ In our scoping review of patient engagement in pharmacy practice research, we developed an initial engagement plan based on our team’s previous experiences engaging with PWLE on a scoping review.^25^^,^ ^26^ We then refined this plan in collaboration with PWLE once the research team was assembled.

### 4.1 Deciding who to engage

Researchers should think carefully about which groups, communities, or individual PWLE could bring meaningful and unique perspectives to their knowledge synthesis. Some important questions to consider are: *Who cares about this research and its results? Who will be affected by the findings? Who has personal experience that could provide valuable insight? Are there voices that are often overlooked but should be included?* When recruiting PWLE, the goal should be to bring in perspectives from those directly impacted by the research.^24^

In our scoping review, we sought to engage PWLE who had navigated the Canadian healthcare system, either as patients or care partners. We chose to recruit PWLE from across Canada to gain a range of perspectives on the state of patient engagement in pharmacy practice research. However, this decision required PWLE to be able to meet virtually, which could have unintentionally excluded some perspectives. Prior experience partnering on pharmacy practice research was preferred, but optional, and prior knowledge synthesis experience was not a prerequisite for engagement. These criteria were chosen with intention, as it is important to create opportunities for PWLE who are new to engagement to engage.^27^ We recruited four PWLE team members to foster peer mentorship and support, which can enhance the engagement experience.^28^

**Figure:**
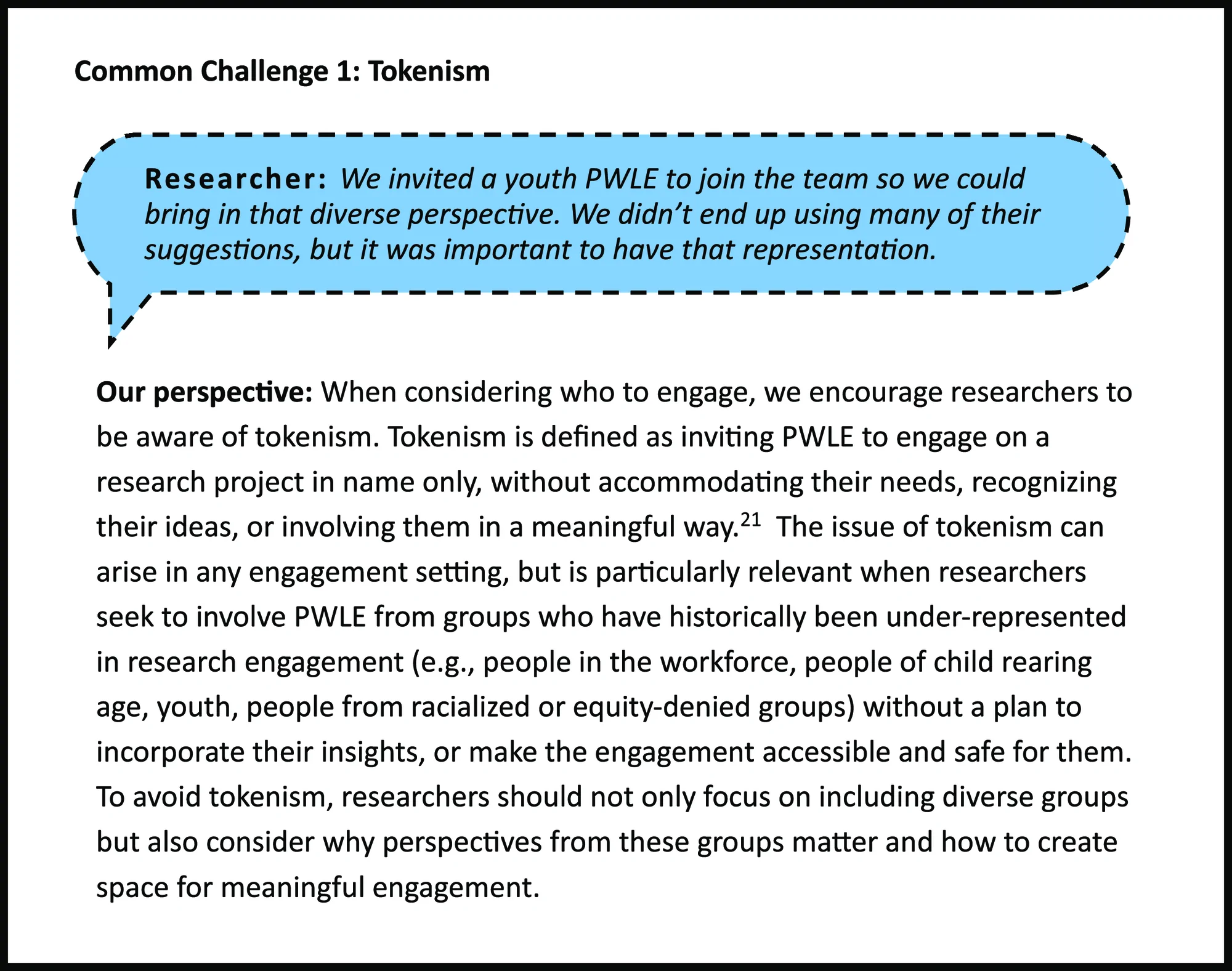

### 4.2 Deciding when to engage

Many PWLE value being involved early in research, during the idea generation stage, when they can help shape its direction before major decisions are made.^20^ Early collaboration also supports one of the central goals of patient engagement: to advocate for research that focuses on priorities and outcomes that matter to PWLE.^9^ When we began working with PWLE, some initial planning had already been completed, including a preliminary protocol for our scoping review of patient engagement in pharmacy practice research. Through PWLE engagement, we aimed to optimize the protocol. We also hoped the PWLE involved would remain engaged throughout the process of conducting the scoping review.

**Figure:**
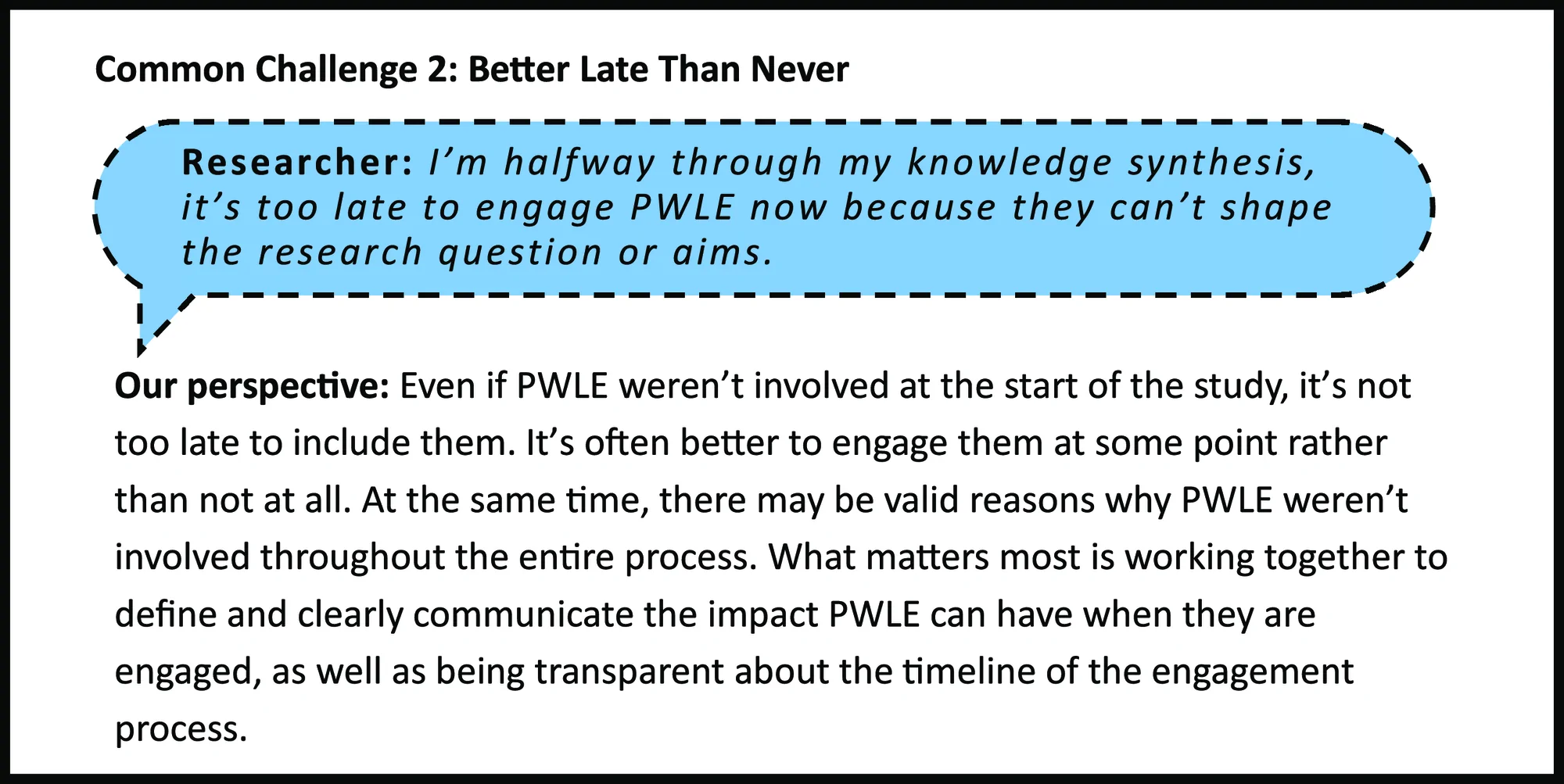

### 4.3 Planning for compensation and reimbursement

Recent studies highlight the importance of compensating PWLE for their time and reimbursing expenses like transportation, childcare, or respite costs.^29^ Before engaging, researchers should assess whether their existing research funding can support this compensation or dedicate specific budget lines toward it when preparing new grant applications. Planning for PWLE compensation should follow existing guidelines to ensure fairness.^23^^,^ ^30^

Ideally, compensation planning is done in partnership with PWLE. However, we recognize that some research teams may not yet be collaborating with PWLE at the proposal stage. In these cases, it is important to revisit the proposed compensation model with PWLE after they have been recruited onto the study team. This helps ensure that everyone agrees with and understands the compensation approach. It is also important to discuss what kinds of compensation might be best suited to the needs of PWLE on the team. Some may prefer an hourly rate, lump sum, or request a donation be made on their behalf. Others may wish to be compensated through access to equipment (e.g., being provided with a laptop) or educational opportunities. With this information in hand, researchers can develop compensation plans that meet PWLE’s needs, while also balancing the policies of the institutions where they work. Having these conversations early is key, as compensation issues take time to resolve and can potentially strain relationships between researchers and PWLE.

In our scoping review, PWLE team members were compensated at a rate of $25 CAD per hour. The compensation rate was clearly communicated from the beginning of the engagement process, and we encouraged PWLE to contact the research team if they preferred an alternative form of compensation. All PWLE on our team were comfortable with the stated compensation, and we agreed on the maximum billable hours for each research task to promote transparency and ensure project sustainability.

A challenge to compensating PWLE was that, under our department’s newly revised regulations, we were required to hire PWLE as staff members. The hiring process created an administrative burden for all team members and delayed the disbursement of compensation. Further, hiring PWLE was not common practice in our department, and we were learning to facilitate this process alongside administrative staff. We now have systems in place to streamline this process in the future.

**Figure:**
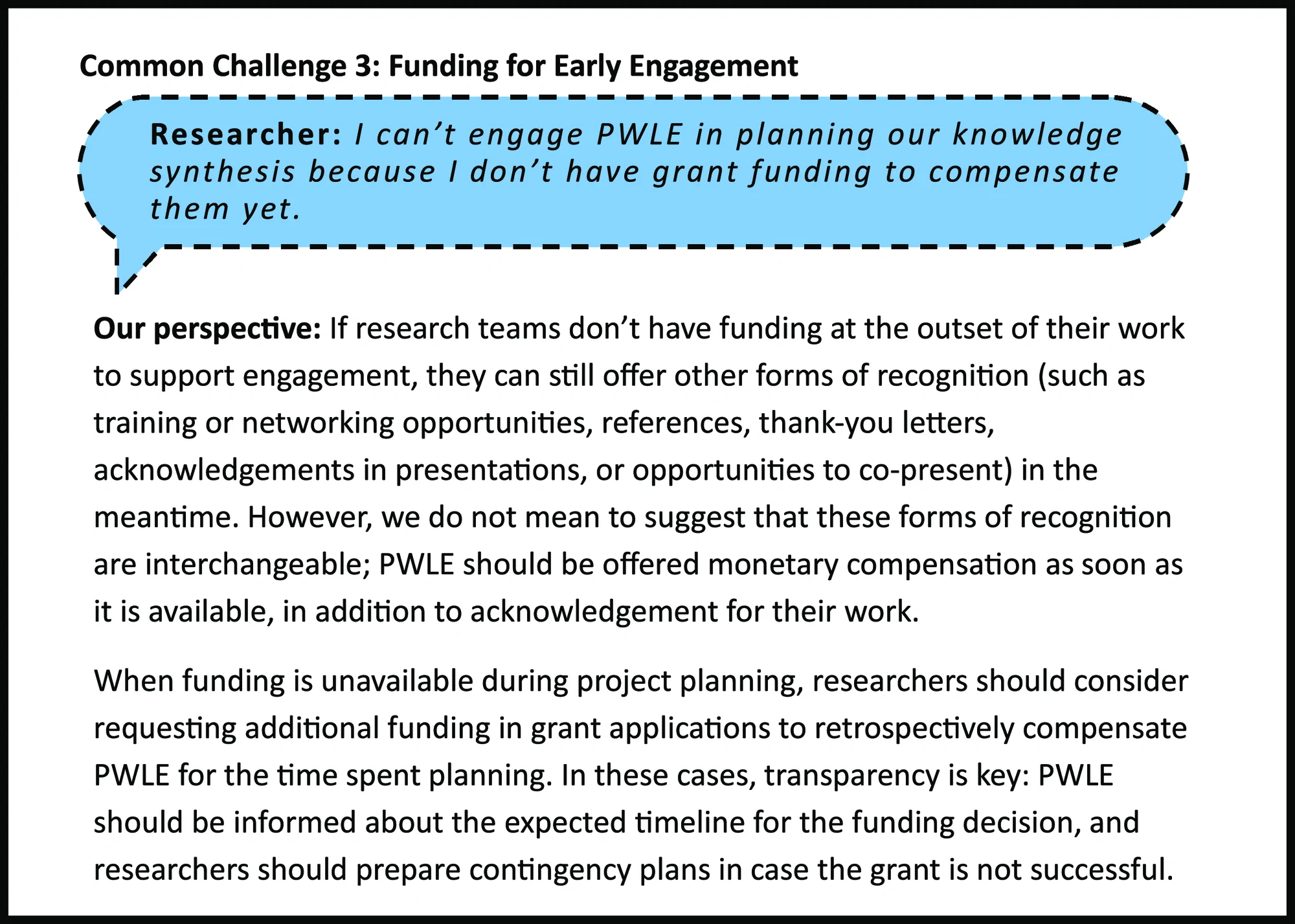

**Figure:**
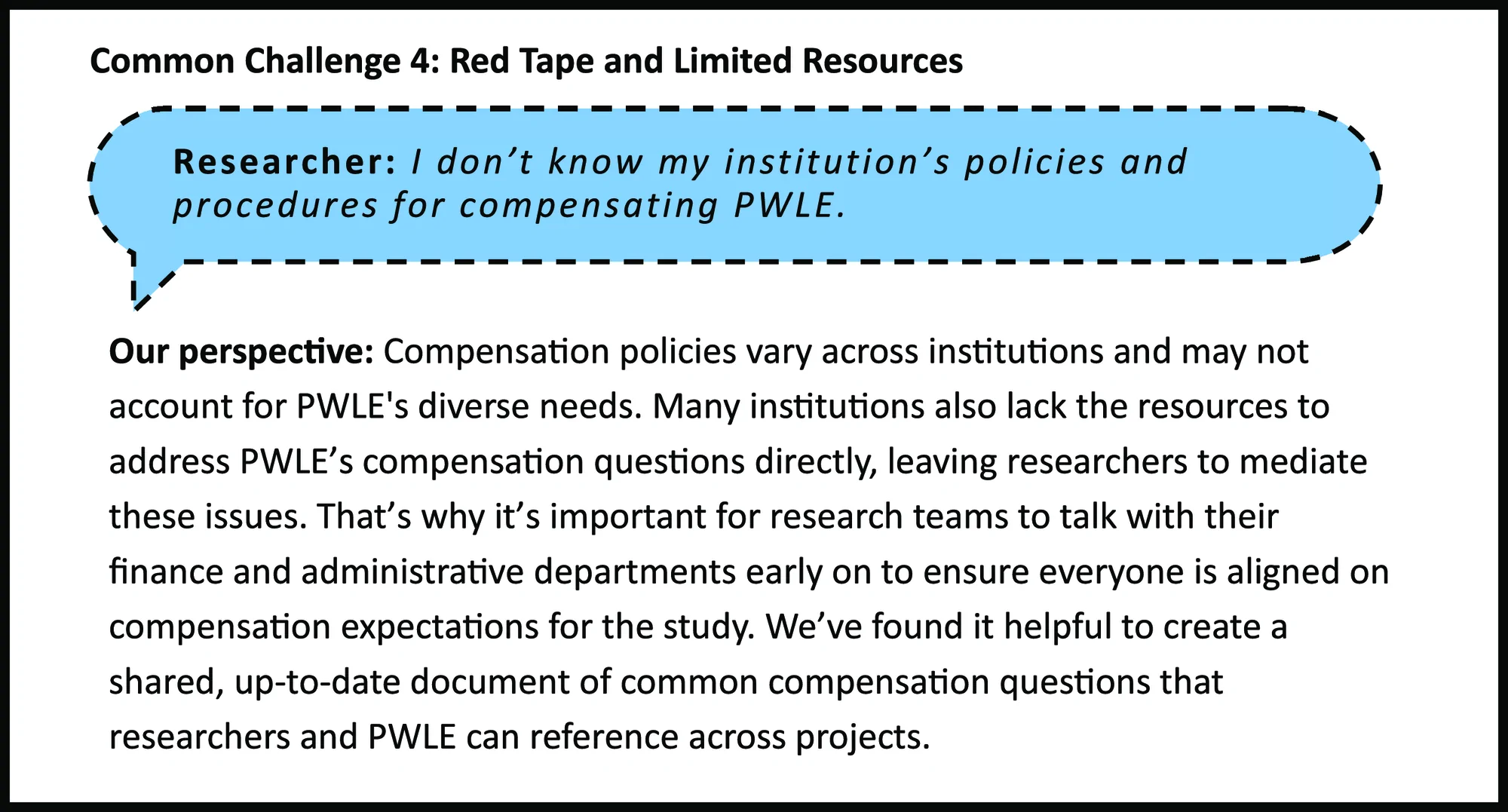

**Figure:**
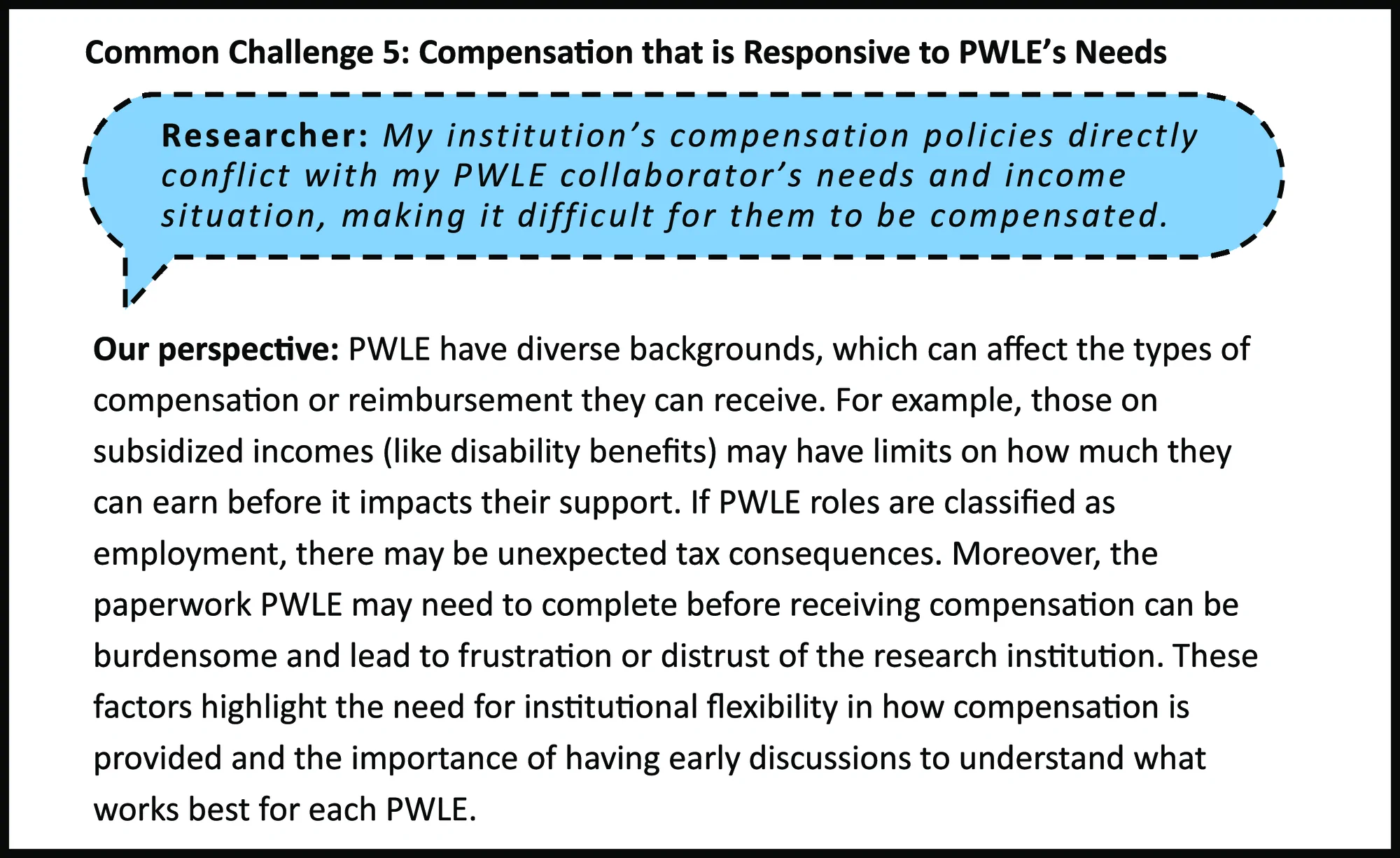

## 5 Stage 2: Recruiting people with lived experience

After the initial planning described above is complete, research teams can move forward with recruiting PWLE who would like to co-conduct the knowledge synthesis.^13^ Key considerations at the recruitment stage include creating materials, promoting the opportunity, and maintaining clear communication with applicants.

### 5.1 Creating recruitment materials

To help PWLE make informed decisions about getting involved, recruitment materials should clearly describe the desired PWLE role, estimated time commitments, activities, any technological requirements (e.g., access to the internet and a webcam), available accessibility supports, and compensation and reimbursement details. Providing this information upfront helps streamline the recruitment process and attracts PWLE who are both interested and able to engage. As an example, Supplementary File 2 contains the recruitment letter we used when inviting PWLE to join our scoping review of patient engagement in pharmacy practice research.

### 5.2 Promoting the opportunity

Research teams should tailor their recruitment strategy to reach the specific groups they want to engage. When recruiting PWLE it is important to take deliberate steps to ensure a diversity of perspectives are represented on the research team. However, in many projects, PWLE tend to represent a narrow demographic group^31^—typically retired, educated, White, middle-class women with prior research engagement experience^32^. This lack of diversity can occur because common recruitment strategies used by researchers (e.g., online advertisements shared through universities or professional networks) may be more likely to reach these individuals.^33^ While all PWLE offer valuable lived experiences to a knowledge synthesis, it is important to create space for diverse groups impacted by the research topic to shape the research directions and outcomes. To reach more diverse or underrepresented groups, researchers should explore targeted advertisement methods, such as distributing physical copies of recruitment materials at local community centers and building trusting connections with community leaders who can help recruit in culturally appropriate and accessible ways.^33^

### 5.3 Communicating with applicants

When recruiting PWLE, there may be more applicants than available positions, meaning some will not be selected. To ensure transparency, researchers should clearly explain the selection process. If possible, they should specify how many PWLE they plan to recruit and any criteria they will use to make their selection. In our research, we found it helpful to ask PWLE applicants to complete a brief introductory meeting before joining the team. This meeting helped us learn about their motivations for getting involved, the lived or living experiences they brought to the team, how they typically worked in a group setting, how they might contribute to the diversity of perspectives in the project, and whether they were available to engage in the study activities. When communicating with PWLE applicants, researchers should share details about the recruitment timeline, including the application deadline and when applicants can expect to be notified with the results, regardless of whether they were successful or not. From the PWLE perspective, not hearing back after applying to join a research project (sometimes called “getting ghosted”) can be discouraging and creates hesitation around applying to engagement opportunities in the future.

## 6 Stage 3: Building rapport and establishing the team

The four principles of PWLE engagement endorsed by the Canadian Institutes of Health Research SPOR—mutual respect, inclusiveness, co-building, and support^9^—are only achievable with strong working relationships. At the beginning of a knowledge synthesis, it is important to plan activities that help build team cohesion and foster rapport between all members of the research team.^13^ We describe some of these actions and activities below, as well as how we applied them in our own work.

### 6.1 Designate an engagement liaison

One step research teams can take to promote meaningful engagement is to designate a team member who ensures that the integrity of the engagement process is upheld.^13^ This person, often called a “patient engagement liaison” (or PWLE engagement liaison), focuses on building trust and fostering positive relationships with PWLE, serving as their main point of contact for questions, comments, and concerns. Most importantly, the liaison should advocate for the voice of PWLE in the research process and facilitate communication and the co-creation of PWLE roles. In our research, two team members served as engagement liaisons. As a patient-oriented researcher, AMC oversaw all aspects of engagement in our scoping review. As a research trainee working under AMC, SK later served as the primary point of contact for PWLE during the development of the present article.

### 6.2 Lay the groundwork: Creating a terms of reference

**Figure 2:**
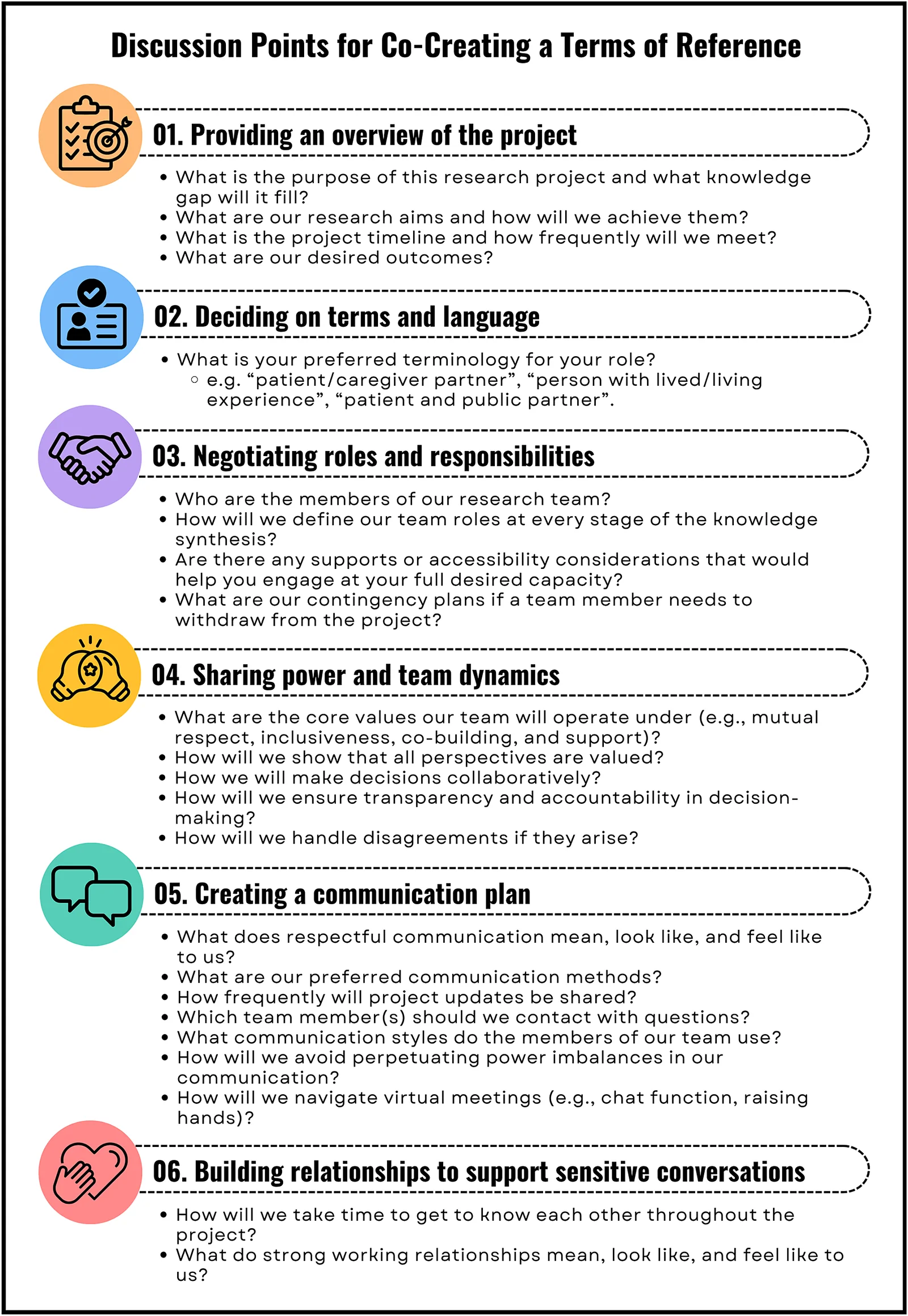
Considerations when co-creating a terms of reference. Figure 2 long description.

To determine whether positive relationships are developing within the research team, the team must define what these relationships mean, look like, and feel like. One effective way to do this is by co-creating a Terms of Reference document, also known as “guidelines of conduct” or a “team charter.”^13^ This document typically outlines the planned workflow and the team’s needs, roles, and expectations around respectful and inclusive collaboration, constructive conflict resolution, and accessibility or accommodations.^14^ The Terms of Reference is meant to be a living document, revisited periodically as relationships evolve throughout the knowledge synthesis. Below and in Figure 2, we outline six considerations that could be discussed within a Terms of Reference Document, based on our own work with PWLE.

#### 6.2.1 Providing an overview of the study

Before creating a Terms of Reference with PWLE, it is important that all team members are grounded in a similar understanding of the purpose, aims, scope, timeline, and desired outcomes of the knowledge synthesis. Opening the terms of reference meeting with a big-picture overview of the study can be helpful for this reason. We also recognize that the specific details of the study might not be determined at this stage, especially if a research team is building a knowledge synthesis from the ground up with PWLE. Still, it is good practice for the team to answer the question “*how did we get here?”* before moving forward with their work together.

#### 6.2.2 Deciding on terms and language

There are differing opinions within the patient and care partner community about preferred terms for their role. Some feel that labels such as “lived/living experience,” “patient,” or “care partner” are limiting and do not fully capture the complexity of their experiences. Others may prefer terms like “persons with lived experience,” or “patients and the public.”^20^ Rather than assuming what people prefer to be called, it is better for the team to establish a shared language together in their Terms of Reference. Through group discussions, we determined that the individuals engaged in our scoping review preferred the term PWLE, as reflected in the language used throughout this article.

#### 6.2.3 Negotiating roles and responsibilities

PWLE cannot engage to their full desired potential when there is a lack of feedback and support from researchers, leading to disengagement.^13^ Creating a Terms of Reference at the start of the study gives every team member an opportunity to cocreate and clearly define their roles and responsibilities. Team roles should be informed by the strengths and preferences of PWLE, the study’s requirements, and available funding to ensure fair compensation.^13^ It can also be helpful to agree on roles based on different stages of the knowledge synthesis and what training and other capacity building efforts are required by each team member. These conversations are especially important when PWLE have had negative past experiences with engagement and may feel hesitant to work with researchers again. It is helpful to address these experiences directly when developing the terms of reference and to reflect on how similar experiences can be prevented in the current project.

Relatedly, it is important to consider how much time is needed for study-related tasks. As the study progresses, teams should track the time PWLE spent on research tasks and eventually publish these details to improve future planning. An additional consideration is the timing of meetings; researchers may need to hold meetings outside of business hours to accommodate the schedules of PWLE. Preferred meeting times should be identified as a group, and alternative participation options for missed meetings should be discussed.

In our scoping review of patient engagement in pharmacy practice research, we utilized the IAP2 Spectrum of Public Participation^22^ to define the roles of PWLE. Specifically, when seeking PWLE’s input on the review protocol, we classified their engagement as “Involve” on the spectrum,^22^ meaning we aimed to incorporate their ideas into the final protocol draft and clearly explain how their input influenced decision-making. When engaging PWLE in the conduct of the scoping review itself, we framed this process within the IAP2 level of “collaborate,”^22^ as PWLE would be involved in scoping review activities and decision-making to the fullest extent they desired throughout the research process. In developing our Terms of Reference, we outlined PWLE’s preferred level of engagement, identified necessary training for engagement, and discussed contingency plans for missed meetings (e.g., one-on-one meetings, watching recordings of the group meeting, and providing written input on what was discussed).

#### 6.2.4 Sharing power and team dynamics

Sharing power in a research team requires a deliberate effort to move beyond traditional academic hierarchies and to value the perspectives that all team members bring.^9^^,^ ^13^^,^ ^20^ In the Terms of Reference, researchers and PWLE should collaborate to clearly define how decisions will be made within the team and how PWLE will influence the knowledge synthesis at different stages of the research process. An engagement liaison can help facilitate this power-sharing by advocating for the PWLE voice and ensuring their input is heard and acted upon. Other processes for intentionally sharing power could include rotating facilitation between researchers and PWLE team members, inviting PWLE to lead agenda items, and encouraging the entire team to be vocal and identify when processes need to change.

In our work, we shared power by clearly documenting and discussing, as a group, how PWLE feedback was incorporated into the scoping review protocol and the development of the present article. To facilitate this approach and ensure PWLE input actively shaped the research, we designated a member of the academic research team as the note-taker during each meeting. As all of our meetings were conducted via videoconferencing, this person shared their screen with the entire team and took notes on PWLE feedback and updated study documents in real time. This process allowed PWLE to verify that their comments were correctly understood and see how their feedback was being incorporated. When reflecting on our team dynamics, one PWLE noted that we fostered genuine co-construction and created space for questions and dialogue between researchers and PWLE as the projects evolved.

In any discussion of power and team dynamics, it is also essential to talk about trauma-informed, intersectional approaches to engagement.^34^ PWLE bring not only stories of resilience into research spaces but can also recount experiences of grief, pain, being silenced, or being harmed. This reality must be acknowledged throughout the engagement process. For some, sharing their lived experience can be empowering, but it may also reopen emotional wounds. Importantly, PWLE perspectives are shaped not only by personal experiences but also by their intersecting identities. Factors such as race, ethnicity, ability, gender, socioeconomic status, geography, Indigeneity, and language, among many others, influence how included they may feel. Thus, engagement strategies must be intentionally designed with these intersecting identities in mind.^34^ In our research, we reflected on the prompts provided in the Readiness to Engage Workbook^35^ supplied by our local CIHR SPOR SUPPORT organization to reflect on our positionality as a team before engaging with PWLE.

**Figure:**
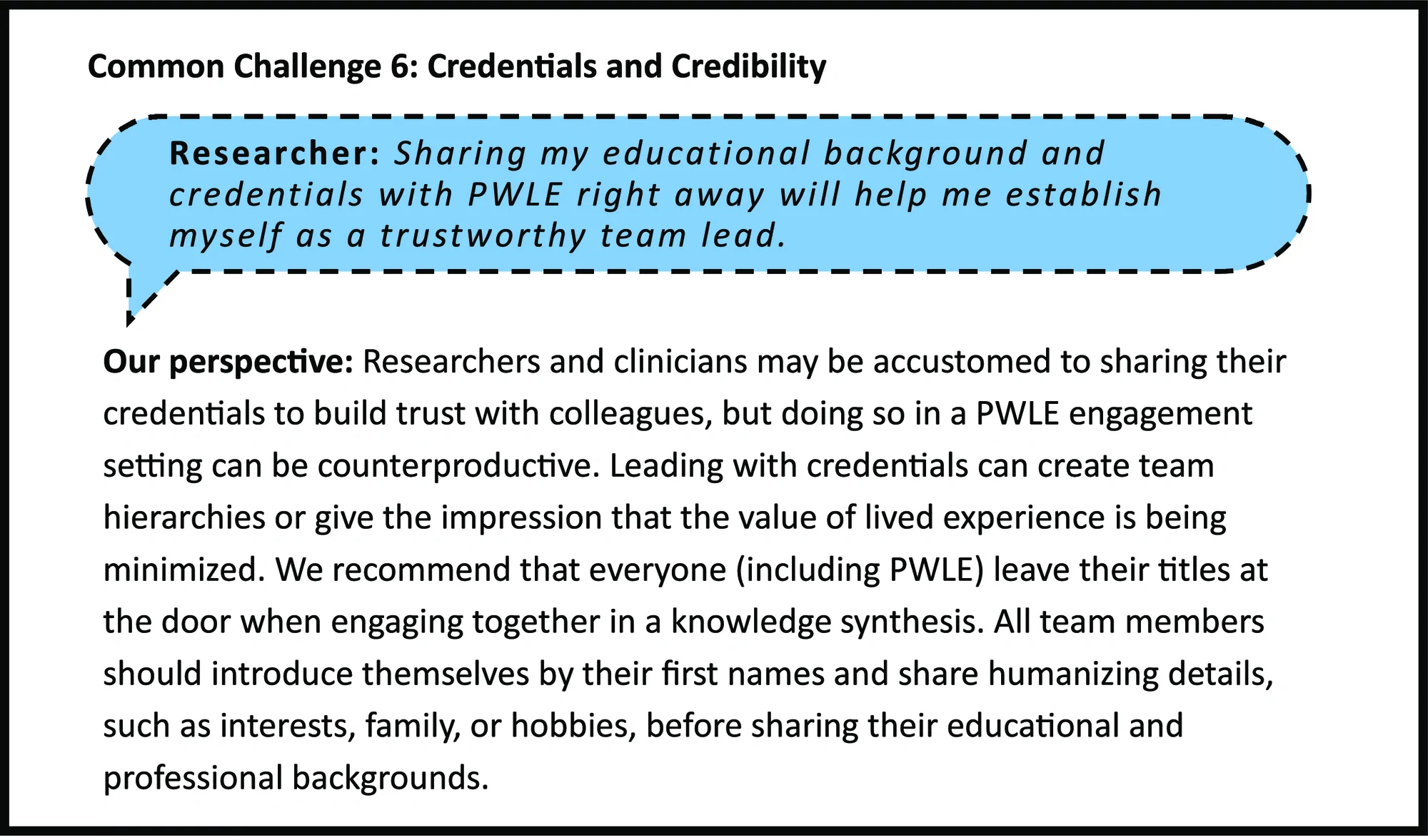

#### 6.2.5 Creating a communication plan

PWLE and other research team members often have different expectations about communication methods, frequency, and what they consider respectful dialogue.^13^ The Terms of Reference should define how often study updates are shared with the group and how these updates are communicated (e.g., via email or meetings). The team should also establish standards for respectful dialogue and develop approaches to handle group conflicts that are mindful of potential power imbalances, ensuring all members feel safe to share their perspectives. Additionally, with virtual meetings becoming more common, research teams should discuss their policies on chat functions and how ideas are shared (e.g., whether team members interject or raise their hands) to make sure everyone can effectively engage. During our initial meetings with PWLE team members, we discussed their preferred communication methods and mutually agreed that email and Zoom videoconferencing were acceptable. In our first group session, we collaboratively established guidelines for respectful communication during videoconferencing.

**Figure:**
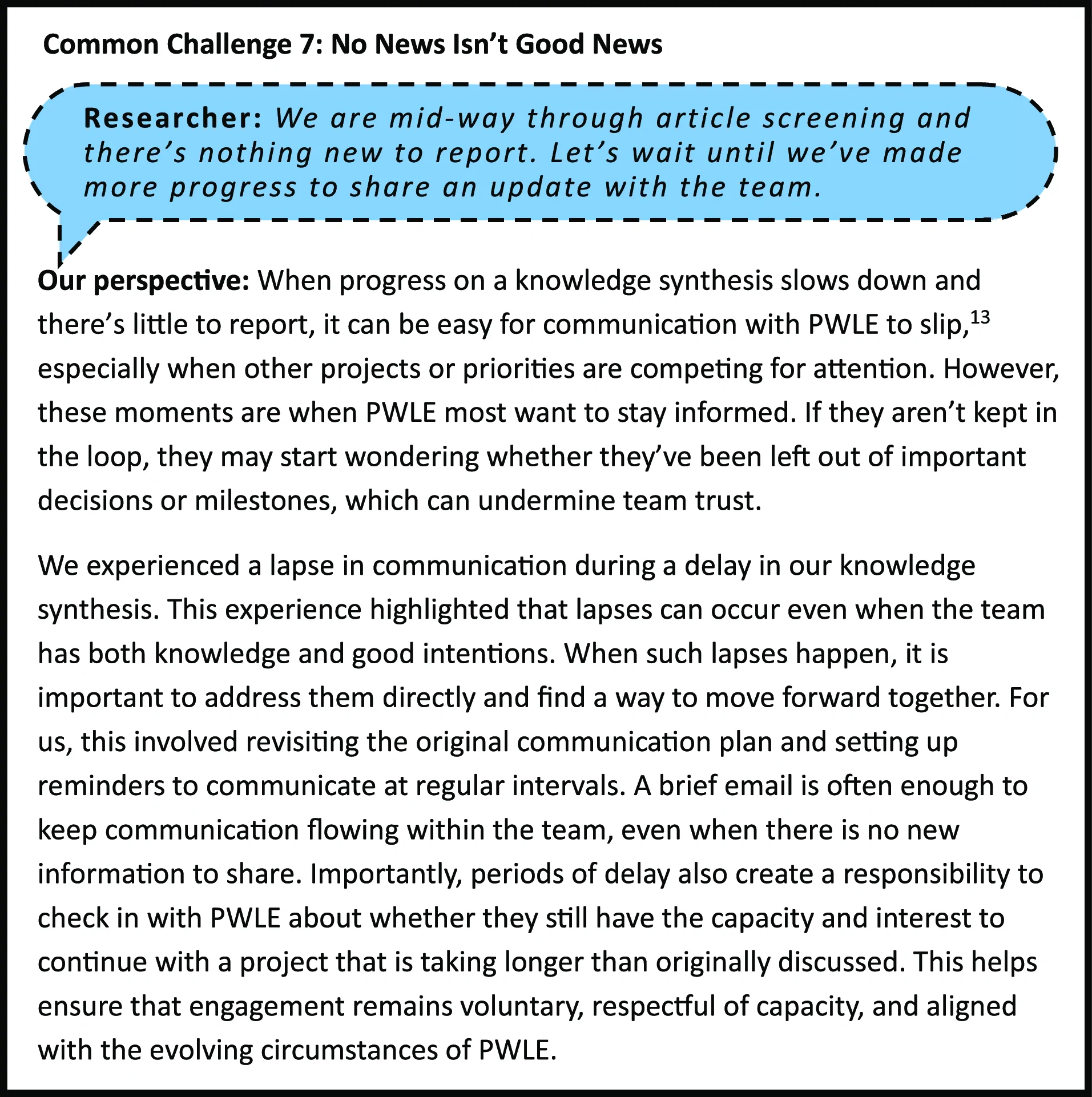

#### 6.2.6 Building relationships to support sensitive conversations

To inform a knowledge synthesis or contextualize their perspectives, PWLE may choose to recount personal experiences as patients or care partners. Building strong relationships within the research team is key to creating safe spaces for PWLE to share these sensitive stories.^24^ In a Terms of Reference, researchers and PWLE can decide how to allocate time to relationship-building throughout the knowledge synthesis. In our research group, we typically reserved the first 10 to 15 minutes of our meetings for informal check-ins and incorporated some informal discussion throughout each meeting. Other teams may choose to share meals together (if working in person) or have informal “coffee calls” throughout a project. From the PWLE perspective, when members of a research team know each other on a personal level, sharing their insights and experiences feels easier and safer.

## 7 Stage 4: Building capacity for engagement

Capacity refers to an individual or group’s ability to complete a task given available skills, abilities, and resources. Successfully co-conducting a knowledge synthesis requires capacity building for both PWLE and researchers alike. When PWLE do not receive the tools or support needed to engage meaningfully in a knowledge synthesis, they may feel frustrated or unable to express their opinions or concerns.^13^ When researchers do not receive the training needed to meaningfully engage PWLE, they may encounter roadblocks in the project or challenges within the team dynamic. As described below, research teams should consider the training needs of all team members when embarking on a knowledge synthesis that incorporates PWLE engagement and the impacts of training should be evaluated regularly.^13^

### 7.1 Building capacity among PWLE

PWLE are a diverse group with experiences that go beyond just their lived and living experiences of health conditions or interactions with the healthcare system. Like the rest of the research team, they may also have different levels of experience with knowledge syntheses.^13^ Instead of assuming what everyone knows, it can be helpful to provide the same resources and training to the whole group. This ensures no one feels singled out for knowing less and gives everyone the same opportunity to build on their existing knowledge. Given that the PWLE members of our team had varying levels of experience with knowledge syntheses, our group found that workshops focused on skill development boosted confidence and competence for contributing to the knowledge synthesis. Offering educational materials in various formats, such as videos and written documents, also helped accommodate multiple learning styles.

Building capacity among youth PWLE is especially important, as they are emerging leaders in patient advocacy and may be engaging in research projects for the first time. From the perspective of the youth PWLE on our team, youth face unique pressures, such as navigating the healthcare system at a young age, balancing school or caregiving responsibilities with engagement, voicing their thoughts among adults who may speak over them, and feeling the need to represent their generation as the youngest voice at the table. To support youth PWLE effectively, it helps to involve more than one youth whenever possible, schedule dedicated check-in sessions to foster relationship-building and skill development, collaborate with a youth-friendly patient engagement liaison, avoid jargon, and ensure communication and activities are tailored to youth. Although we engaged only one youth PWLE on our research team, appointing a PhD student as the PWLE engagement liaison, who was similar in age, helped facilitate strong communication and relationship-building.

**Figure:**
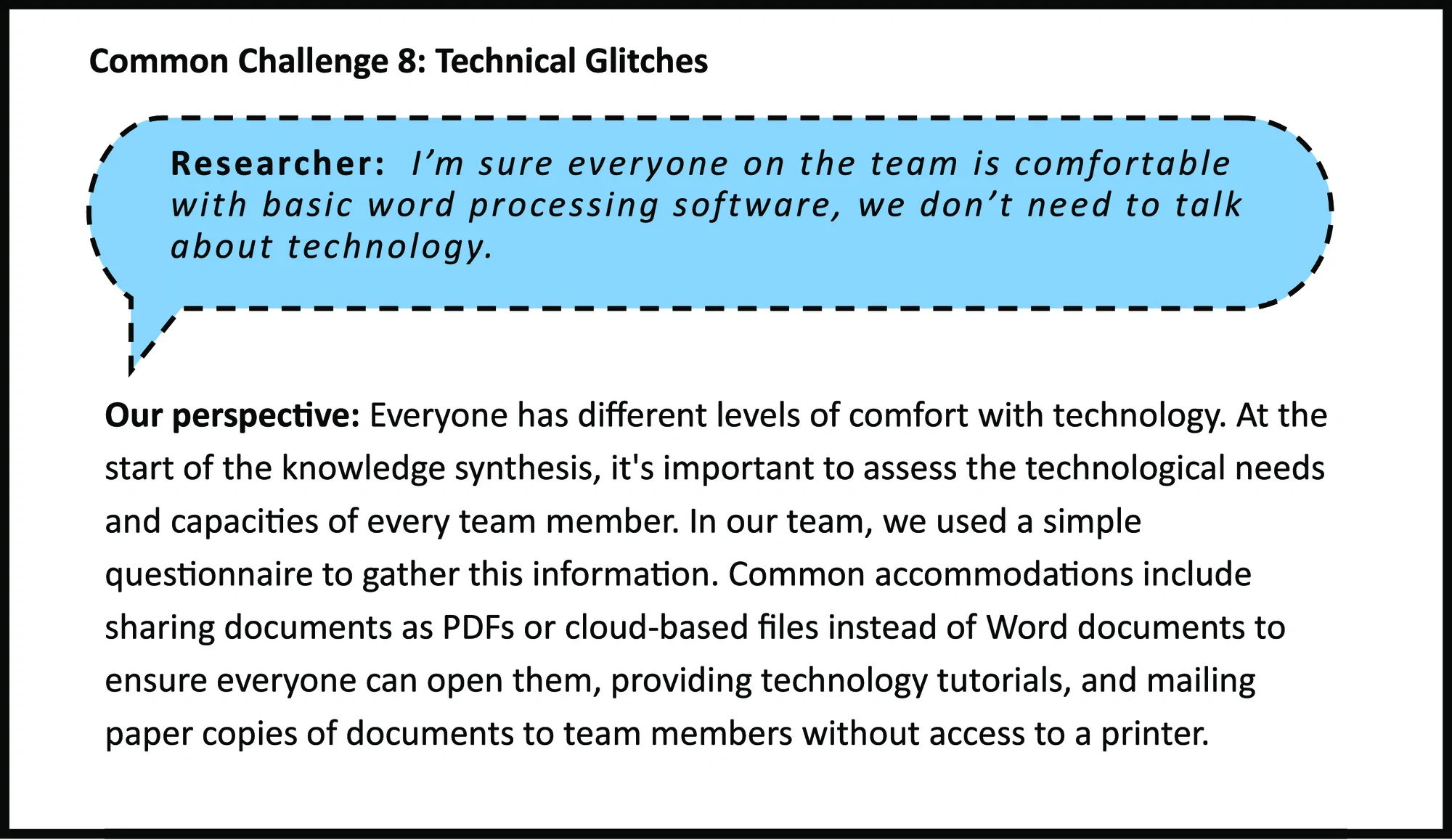

### 7.2 Building capacity among researchers

Education and training should not be limited to PWLE; researchers also need the skills and resources to engage PWLE meaningfully and effectively.^13^ For example, a team might seek training on how to create culturally safe, trauma-informed engagement experiences,^34^ take a course on PWLE engagement, or read articles (like this one) on how to engage PWLE in different research settings. From the PWLE perspective, facilitating engagement is a skill that develops with practice; the more researchers engage, the more confident and effective they become. Researchers, especially those new to engagement, should remember that mistakes are part of the learning process and what matters most is being open to receiving feedback and improving over time. Within our team, SK completed a graduate-level course on patient engagement in research taught by AMC. SK then received experience-based training as the PWLE engagement liaison in preparing this article. The skills SK gained through these experiences will be carried forward to future PWLE engagement initiatives as part of an ongoing learning process.

### 7.3 Evaluating the impact of training

When the training resources shared with PWLE are publicly reported and evaluated, future capacity-building efforts can be improved.^13^ However, there is limited guidance on which resources are helpful for researchers and PWLE engaging on a knowledge synthesis together. The field would benefit from research teams sharing training materials and evaluations of these materials alongside academic publications or other research outputs. Although our team did not formally evaluate our capacity-building resources, we have included the materials offered to PWLE in Supplementary File 3. In addition, we provided PWLE with key articles related to our knowledge synthesis topic: patient engagement in pharmacy practice research.

## 8 Stage 5: Conducting the review

The way a research team approaches engagement plays a key role in building a shared vision with PWLE. From the outset, it is essential to create a safe and welcoming space where PWLE feel comfortable sharing their ideas and actively participating. Effective engagement during the planning and execution of a knowledge synthesis involves setting clear expectations, offering meaningful opportunities to contribute, and establishing realistic timelines that respect everyone’s capacity and commitments.^13^ Our scoping review is currently in the data analysis phase. Consequently, our insights on analyzing data and mobilizing findings are based on existing literature,^13^ our prior research experiences,^25^^,^ ^26^ and our future plans for collaboration.

### 8.1 Managing expectations

Researchers and PWLE may initially have different expectations about the scope of the knowledge synthesis and what it can achieve.^13^ In our own work, we faced an expectation mismatch when we received feedback from PWLE that it was unclear why our scoping review focused only on patient engagement in pharmacy practice research (defined as research about pharmacy practice or pharmacist-led care), rather than on all types of pharmacy research. To address this, we held a group discussion on the need to balance comprehensiveness with specificity in designing a knowledge synthesis research question. Through this conversation, we reached alignment on the review’s scope. Collaborating as a team to discuss and renegotiate the scope of a knowledge synthesis to balance enthusiasm with feasibility has been discussed in a previous review of patient engagement in knowledge syntheses.^13^

**Figure:**
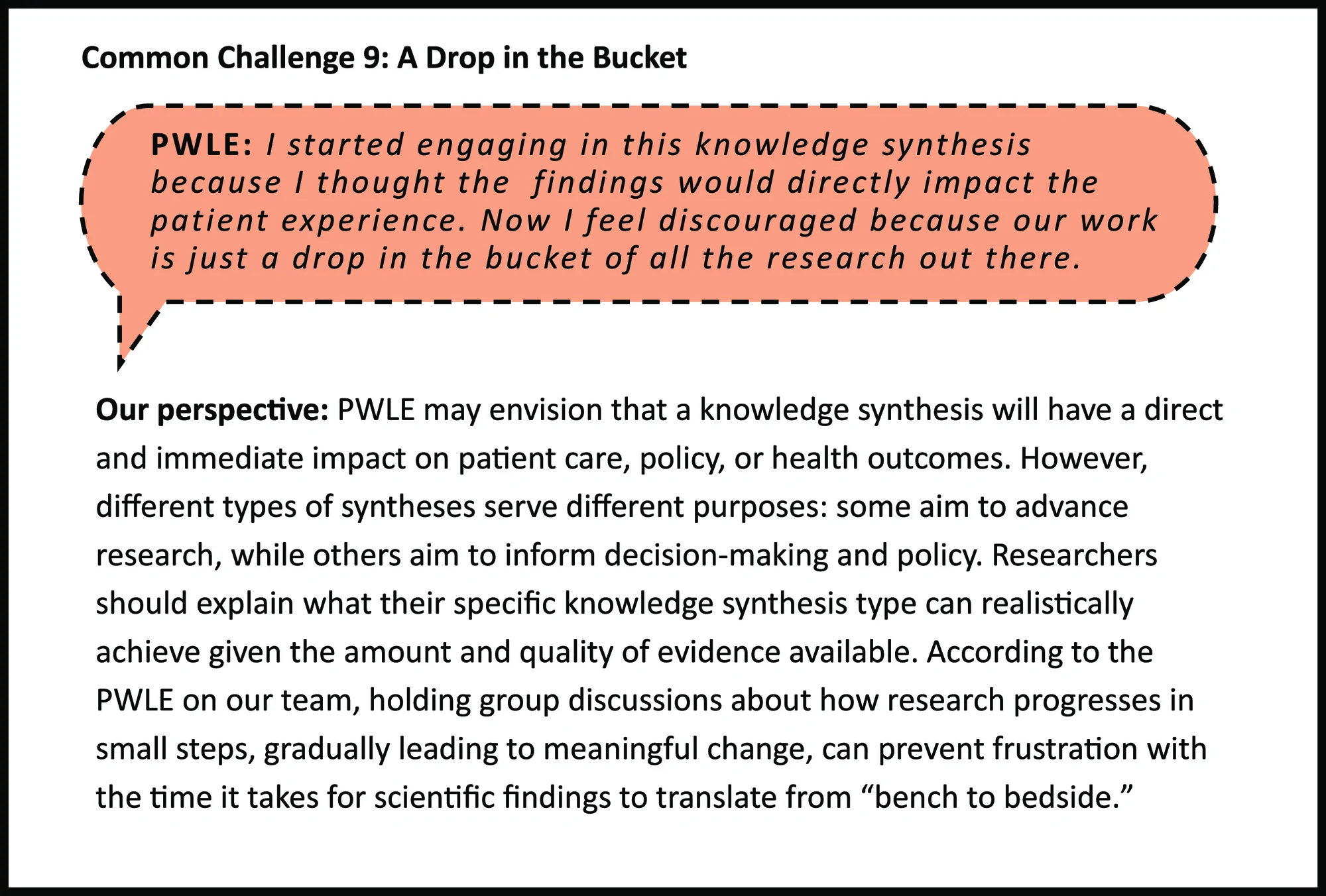

### 8.2 Creating and tracking meaningful engagement opportunities

**Figure 3:**
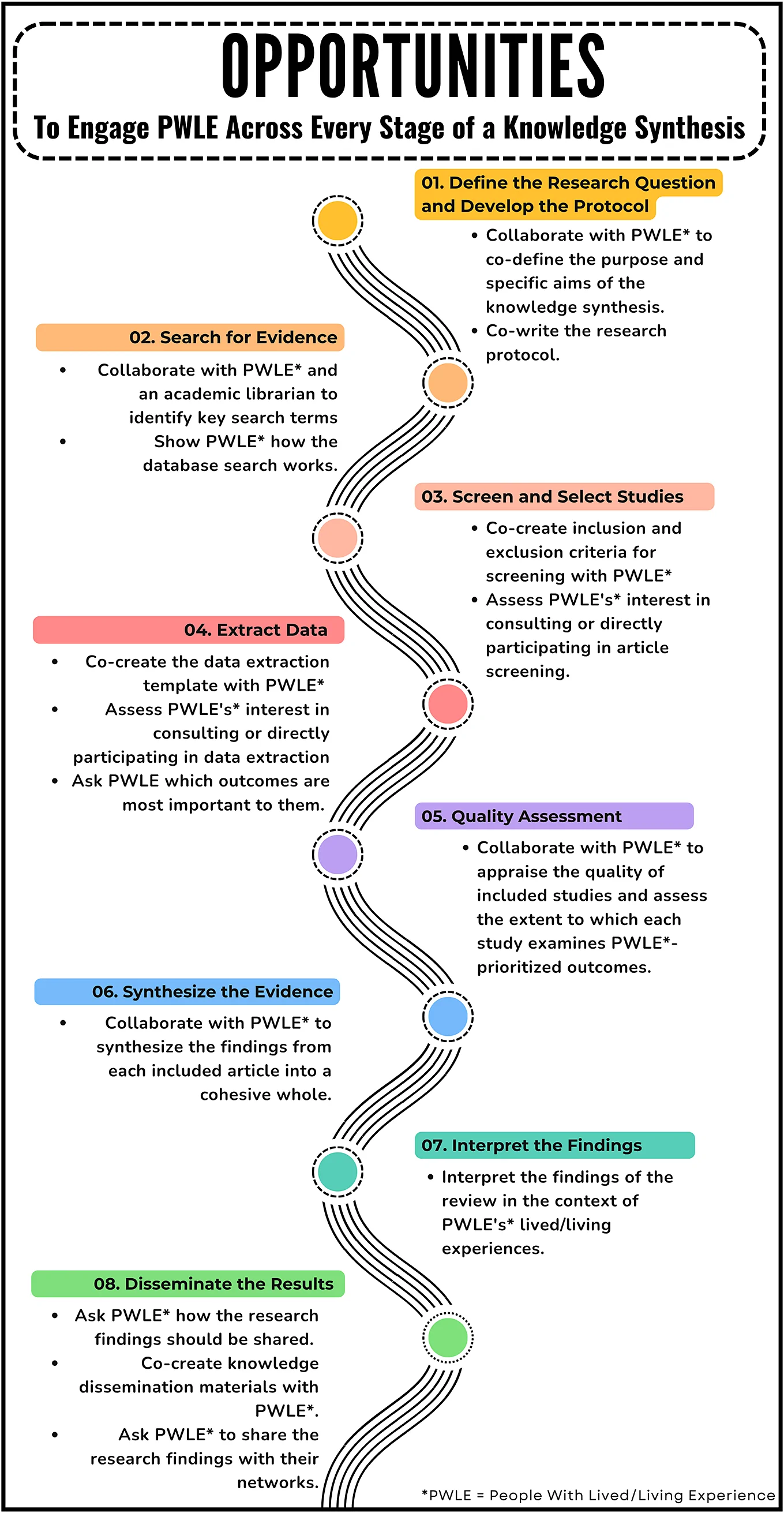
Opportunities to engage PWLE throughout a knowledge synthesis. Figure 3 long description.

Researchers have voiced concerns about limited opportunities for PWLE to shape knowledge syntheses.^13^ Similarly, PWLE might feel uncertain about how their input will actually influence the research.^13^ These concerns can be addressed by following the engagement plans set out in the Terms of Reference while maintaining an open dialogue with PWLE. In our work, we identified opportunities to engage PWLE in every stage of the knowledge synthesis, as described in Figure 3.

In our scoping review of patient engagement in pharmacy practice research, PWLE co-defined the review’s purpose and objectives and co-developed the protocol, including the search terms and screening criteria. The PWLE on our team served in an advisory capacity during article screening and data extraction due to the volume of articles being processed. Nonetheless, we shared information about these processes, including a tutorial on using the Covidence platform (https://www.covidence.org/) for screening articles and managing data extraction. Although we have not yet completed the data analysis stage, as co-established in our terms of reference document, we plan to collaborate with PWLE on interpreting and synthesizing findings, and disseminating the review’s results.

### 8.3 Promoting accessibility

A key role for PWLE engaged in knowledge syntheses is providing feedback on research plans and study documents. Asking for written feedback is common in research, but some PWLE may prefer other ways of giving feedback that better suit their comfort or accessibility needs—taking into consideration digital literacy, sensory safety, cognitive load, and screen fatigue. Researchers and PWLE should decide on their preferred ways of providing feedback together.^13^ In our work, we shared both editable text files and PDFs, allowing team members to choose the format most accessible on their devices. PWLE also had the option to provide feedback directly within the documents, send an email with comments, or schedule an individual meeting with the patient engagement liaison to share their input verbally. We also found it useful to share a checklist of accommodation options with PWLE at the research outset so we could identify helpful supports together (see Supplementary File 4). PWLE emphasized the importance of ensuring all team members—including researchers—could work meaningfully together without having to mask or apologize for their needs.

### 8.4 Establishing timelines

#### 8.4.1 Timelines for people with lived experience

PWLE report time-related barriers to engaging in knowledge syntheses such as projects taking longer than anticipated, unrealistic deadlines, inadequate time to prepare for meetings and provide input, and the time-consuming nature of projects leading to a loss of interest in the work.^13^ We experienced some of these barriers in our own work.

A challenge our team faced was that the timeline for our pharmacy practice scoping review extended beyond our initial expectations. Based on previous experience, we expected that descriptions of engagement, or whether it even occurred, would be missing from the vast majority of article abstracts. As a result, we screened the full texts of all identified articles, which was even more time-consuming than we initially projected. Further, after applying exclusion criteria, we included substantially more articles than we expected based on previous reviews of patient engagement in pharmacy practice research.^36^^,^ ^37^ This delay necessitated ongoing communication to explain the situation and plan for next steps. Fortunately, the delay also allowed us to continue collaborating with PWLE and resulted in the development of the present article. Although our collaboration was extended, it provided us with a valuable opportunity to advance the practice of patient engagement in knowledge syntheses.

Practices that helped us respect PWLE’s time included establishing regular, predictable meetings during the most active phases of the knowledge synthesis, being mindful of time zone differences, using scheduling polls, and working together to set guidelines on the maximum time to be spent on various study tasks. A lesson we have learned through engagement is to ask, rather than assume, about PWLE’s preferences for meeting times and their competing responsibilities (e.g., work, volunteering, childcare, health appointments). If schedules vary, offering multiple meeting options (e.g., weekday and weekend) or recording meetings for asynchronous participation can help ensure inclusive engagement. In our project, asynchronous options were available when PWLE could not attend full group meetings.

**Figure:**
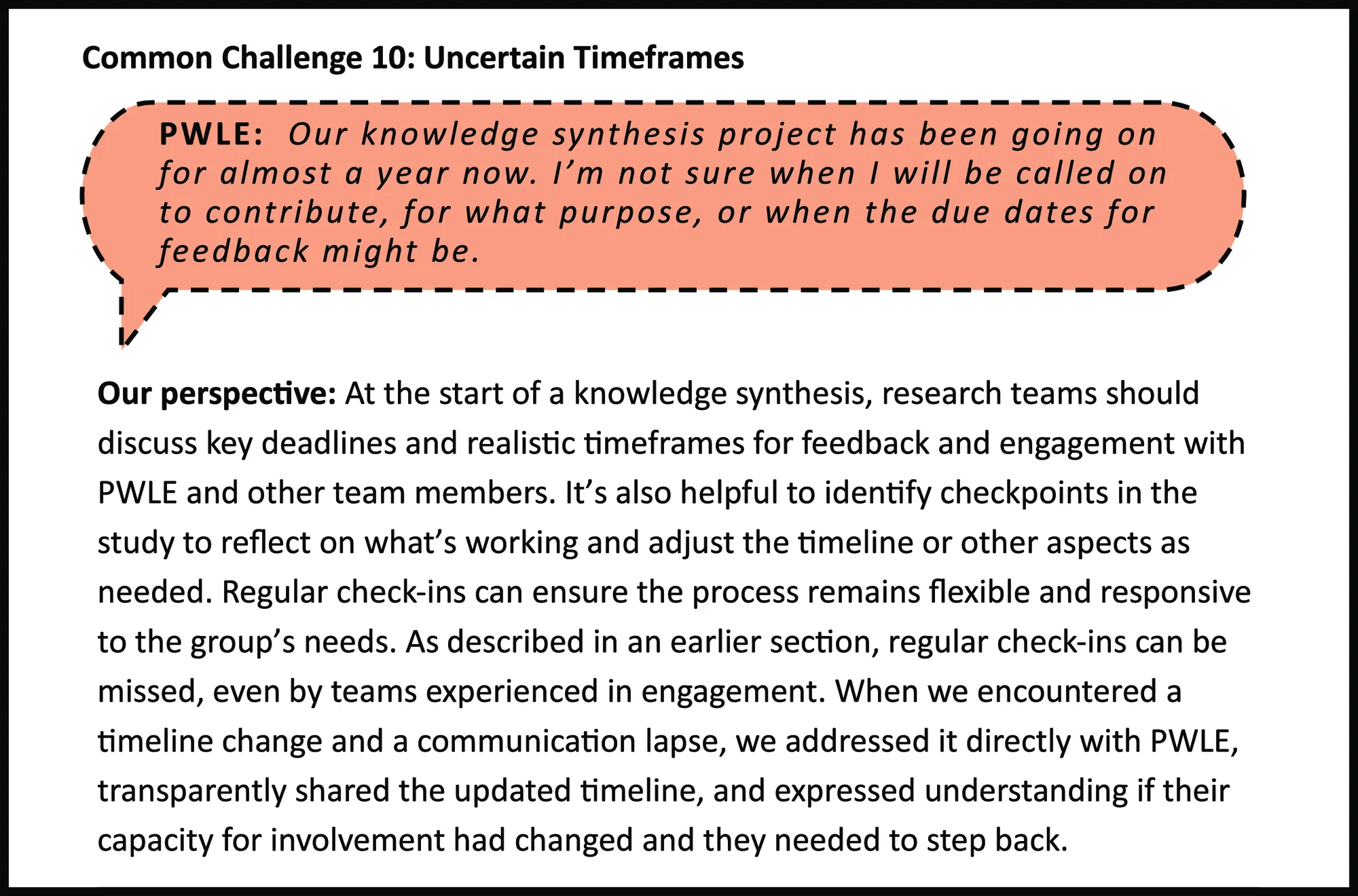

#### 8.4.2 Timelines for researchers

Knowledge syntheses involving PWLE may take more time from conceptualization to publication compared to those conducted solely by researchers. One time-related barrier for researchers includes having few opportunities to build rapport with PWLE and plan meaningful engagement throughout the knowledge synthesis. For some, interactions with PWLE are often confined to the busy phases of the project, which can make relationships feel rushed.^13^ Engaging PWLE in more complex tasks, such as data synthesis, can also be challenging due to the time demands of training and compiling feedback from multiple team members.^13^ In our scoping review of patient engagement in pharmacy practice research, we found it helpful to establish realistic and flexible timelines that accounted for training, relationship-building, and iterative feedback cycles. It was also helpful to appoint an engagement liaison to provide dedicated support to PWLE throughout the project. Ultimately, the additional time required to engage PWLE is an investment in conducting a review with enhanced quality, relevance, and potential for translation into practice.^13^

**Figure:**
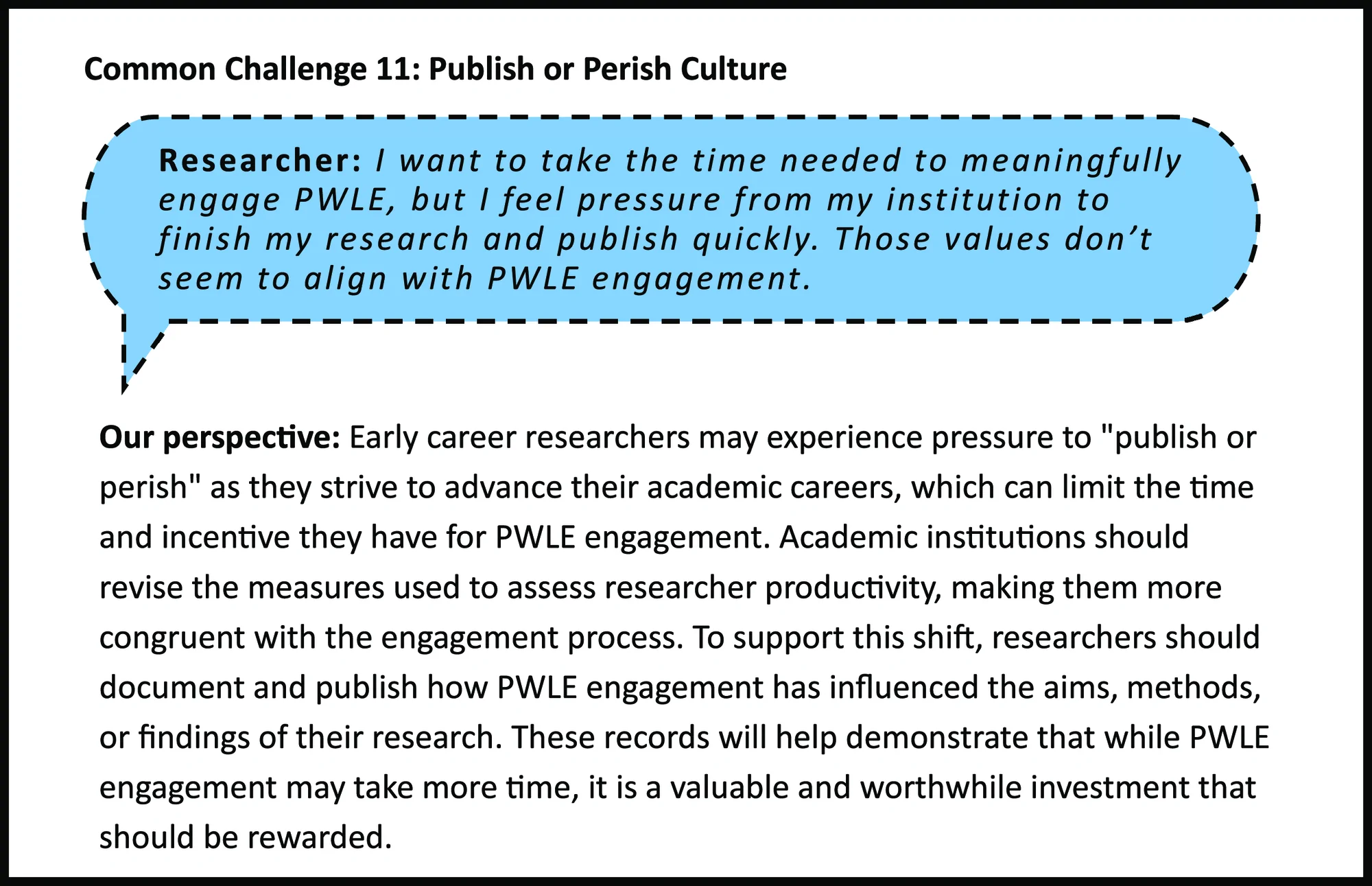

## 9 Stage 6: Mobilizing findings

At the conclusion of the knowledge synthesis, the research team has produced a set of findings that they hope will influence future research, decision-making, policy, clinical practice, or other real-world outcomes. For these outcomes to be realized, the findings must be effectively shared with the world and with key knowledge user groups. PWLE can play a crucial role in mobilizing knowledge by leading the creation of plain-language summaries and reports tailored to specific audiences, sharing research findings within their networks, and helping connect decision-makers and practitioners with the research.^13^ To maximize impact, knowledge mobilization should be planned from the outset of the review and integrated throughout each stage.^2^

**Figure:**
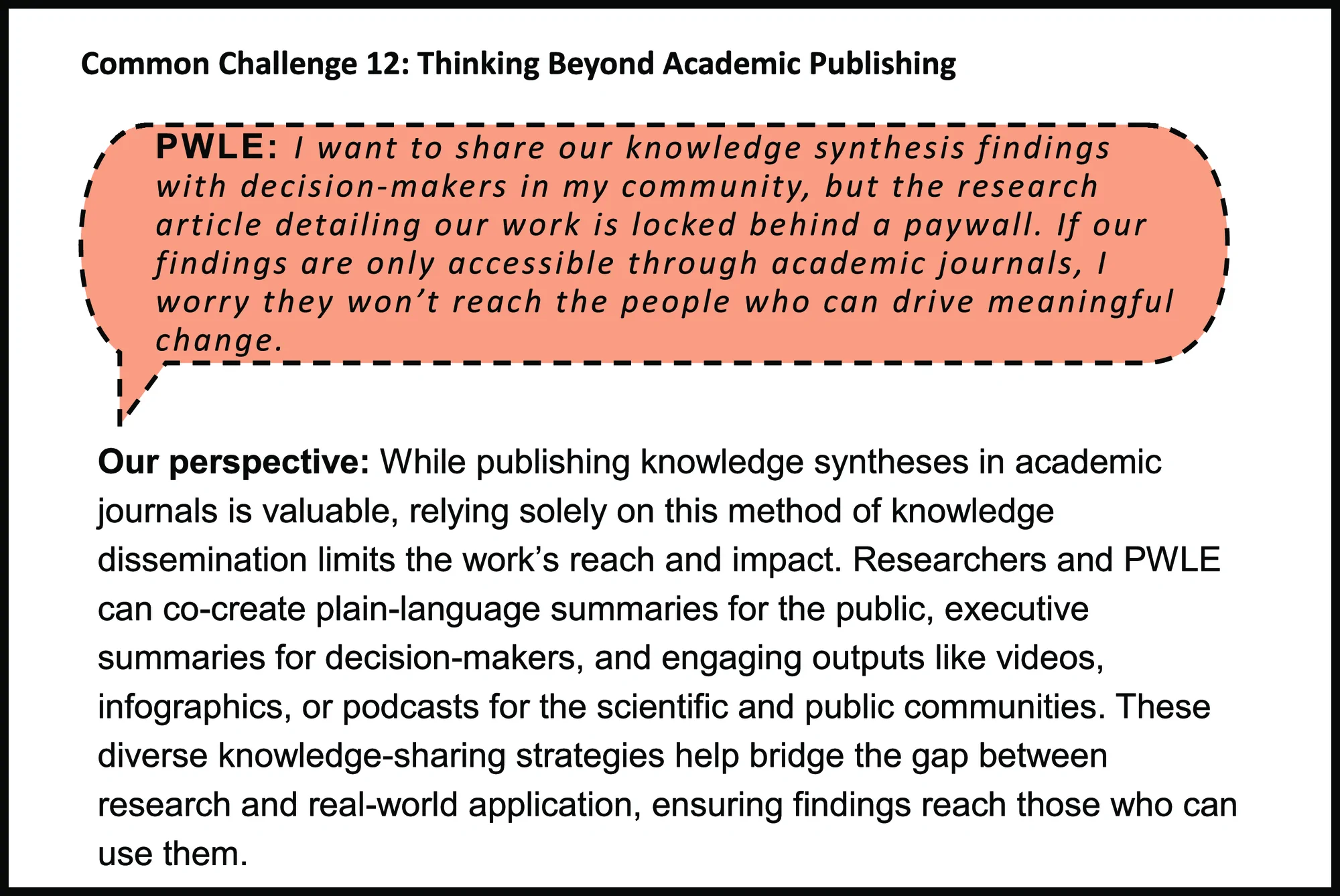

Although our scoping review is still in progress, we discussed knowledge mobilization plans when co-creating our terms of reference with PWLE. These plans included academic publications, infographics that educate researchers on how to engage with PWLE, and infographics for PWLE outlining how they can be engaged in pharmacy practice research. We will continue these conversations as the project progresses, including collaboration with PWLE to identify additional creative knowledge mobilization outputs, as well as with patient and family engagement networks where the findings of the scoping review can be shared.

## 10 Concluding thoughts

In this tutorial paper, we have drawn on the collective experiences of four PWLE, two patient-oriented researchers, two research trainees, and one academic librarian to highlight both the opportunities and challenges of meaningfully engaging PWLE in knowledge syntheses. By working together, research teams and PWLE can synthesize knowledge to address real-world priorities and have a meaningful impact on patients, care partners, clinicians, decision-makers, and the broader research, practice, and policy landscapes. This all starts with the decision to engage, to say an enthusiastic “yes!” to working together. We hope our article serves as a valuable resource in making that decision more accessible and actionable.

## Supporting information

10.1017/rsm.2026.10099.sm001 Chudyk et al. supplementary material Chudyk et al. supplementary material

## Data Availability

There is no dataset associated with this tutorial paper. However, our supplementary materials contain the key documents that our research team used to engage with PWLE. These materials have been provided as examples upon which future research teams can build.

## Supplementary material

To view supplementary material for this article, please visit http://doi.org/10.1017/rsm.2026.10099.

## Long descriptions

### Figure 1 Long description

Circular infographic titled “Meaningful and Active Engagement of PWLE in knowledge syntheses.” At the center is the title inside a circle. Surrounding it are six color‑coded steps connected in a loop. Step 1 is “Create an Engagement Plan,” including deciding who and when to engage and planning compensation. Step 2 is “Recruit People With Lived/Living Experience,” including creating recruitment materials, promotion, and communication with applicants. Step 3 is “Build Rapport and Establish the Team,” including designating an engagement liaison and co‑creating team terms of reference. Step 4 is “Build Capacity for Engagement,” including identifying training needs, sharing resources, and evaluating training impact. Step 5 is “Conduct the Review,” including managing expectations, creating meaningful engagement opportunities, tracking engagement, and establishing timelines. Step 6 is “Mobilize Findings,” including engaging PWLE in identifying and sharing ways to mobilize review findings with leaders and decision‑makers. A footnote explains that PWLE means People With Lived/Living Experience.

Navigate back to Figure 1.

### Figure 2 Long description

The infographic contains six sections.

01. Providing an overview of the project. Icon: Clipboard with a target. Questions include the purpose and knowledge gap, research aims and methods, project timeline and meeting frequency, and desired outcomes.

02. Deciding on terms and language. Icon: Person profile with a checkmark. Questions focus on preferred terminology for roles, such as patient/caregiver partner, person with lived/living experience, or patient and public partner.

03. Negotiating roles and responsibilities. Icon: Handshake. Questions cover research team membership, defining roles at every stage of knowledge synthesis, accessibility supports for engagement, and contingency plans for member withdrawal.

04. Sharing power and team dynamics. Icon: Two hands holding a star. Questions address core values like mutual respect and inclusiveness, valuing all perspectives, collaborative decision-making, transparency, accountability, and handling disagreements.

05. Creating a communication plan. Icon: Two speech bubbles. Questions explore the meaning of respectful communication, preferred methods, update frequency, contact persons, communication styles, avoiding power imbalances, and navigating virtual meetings.

06. Building relationships to support sensitive conversations. Icon: Hand over a heart. Questions focus on taking time to get to know each other and defining what strong working relationships look and feel like.

Navigate back to Figure 2.

### Figure 3 Long description

A central winding path consisting of five parallel lines connects eight colored circular nodes. A footer note defines P W L E as People With Lived/Living Experience.

01. Define the Research Question and Develop the Protocol (Yellow). Bullet points: Collaborate with P W L E to co-define the purpose and specific aims of the knowledge synthesis; Co-write the research protocol.

02. Search for Evidence (Orange). Bullet points: Collaborate with P W L E and an academic librarian to identify key search terms; Show P W L E how the database search works.

03. Screen and Select Studies (Peach). Bullet points: Co-create inclusion and exclusion criteria for screening with P W L E; Assess P W L E's interest in consulting or directly participating in article screening.

04. Extract Data (Red). Bullet points: Co-create the data extraction template with P W L E; Assess P W L E's interest in consulting or directly participating in data extraction; Ask P W L E which outcomes are most important to them.

05. Quality Assessment (Purple). Bullet point: Collaborate with P W L E to appraise the quality of included studies and assess the extent to which each study examines P W L E-prioritized outcomes.

06. Synthesize the Evidence (Blue). Bullet point: Collaborate with P W L E to synthesize the findings from each included article into a cohesive whole.

07. Interpret the Findings (Teal). Bullet point: Interpret the findings of the review in the context of P W L E's lived/living experiences.

08. Disseminate the Results (Green). Bullet points: Ask P W L E how the research findings should be shared; Co-create knowledge dissemination materials with P W L E; Ask P W L E to share the research findings with their networks.

Navigate back to Figure 3.
