# Supplementary material for: Facilitators and barriers to engaging patient partners in knowledge syntheses: A stage-based approach
Source: Res Synth Methods. 2026 Jun 15;17(6):1060–80. doi: 10.1017/rsm.2026.10099 (PMC13732379; doi:10.1017/rsm.2026.10099)
Supplement: Supplementary file 1 [file S1759287926100994sup001.zip › S1759287926100994sup001/Supplemental File 1 Terms of reference - new.docx]

**Terms of Reference for Patient Engagement**

**Brief overview of the project we’re working on:**

The importance of patient engagement in health care and research is becoming increasingly recognized, including its potential to improve care outcomes and research quality. In the field of pharmacy, patient engagement has become increasingly relevant with the evolution of the pharmacist’s role in patient-centred care, medication management, and disease prevention. Yet, despite the emphasis on patient-centred approaches in pharmacy, patient engagement is still uncommon in pharmacy practice research (defined as research about pharmacy practice or pharmacist-led patient care, where the population is pharmacists in a practice setting or the intervention and/or control is being delivered by a pharmacist in a practice setting). This includes, but is not limited to, clinical and outcome research, health services research, and comparative effectiveness research. Therefore, to help guide the growth of patient engagement in pharmacy practice research, we have set out to map the current state of patient engagement in pharmacy practice research.

**Preferred terminology**

**Person with lived experience (PWLE)** instead of patient partner

- Our team’s thoughts: This term is much more inclusive of caregivers and patients and is becoming more commonly used in the engagement literature (important in a way to have a standardized term for “PWLEs” so everyone who reads the literature knows that is being referred to)
- On the IAP2 [spectrum of public participation](https://iap2canada.ca/Resources/Documents/0702-Foundations-Spectrum-MW-rev2%20(1).pdf?__hstc=163327267.476a8eeaad27bb228b9c2403afb983f5.1715177610629.1715177610629.1715177610629.1&__hssc=163327267.2.1715177610629&__hsfp=3815498756&hsCtaTracking=fe26c53d-2dca-4fe7-ac8a-5ffd86b9ffc4%7C05e33fdd-10ed-45ac-bc11-0019045978a5), we will *collaborate* with PWLEs, where decision-making is shared between PWLEs and the rest of the research team. Activities used to engage PWLEs and how accountability for shared decision-making will be upheld are further described below.

**Members of our core patient engagement working group and their positions:**

[Redacted]

**Members of our broader research team and their positions:**

[Redacted]

**General roles and responsibilities for PWLEs:**

- Share lived experiences and other knowledge and ideas with the research team.
- Uphold the confidentiality and privacy of the group.
- Contribute towards a safe, respectful, welcoming, and inclusive environment.
- Contribute to research activities, including attending team meetings.
- Check emails regularly (every few days) for study-related communication.
- Review documents ahead of team meetings.
- Provide timely feedback on manuscripts and other related documents (e.g., abstracts, posters, proposals).
- Let the PWLE liaison know ahead of time if you are unable to attend a meeting or need more time to complete a task.
- Provide feedback on the process and bring up any concerns to the PWLE liaison so that they can be addressed promptly.

**Roles and responsibilities for PWLE liaison:**

- Uphold the confidentiality and privacy of the group
- Contribute towards a safe, respectful, welcoming, and inclusive environment.
- Communicate regularly with PWLEs, including during periods of “research lulls.”
  - This will take the form of regular update emails every 2 weeks since the last meeting or update.
- Listen to and address any questions or concerns promptly.
- Help ensure that PWLEs’ voices are heard and considered equally to all other members of the research team.
- Bring the information that PWLEs share forward as evidence (with their consent) even when they aren’t there.
- Help uphold the preferred characteristics of our working environment (as further described below)

**Roles and responsibilities of the remaining research team members:**

- Value lived experience as a form of knowledge.
- Use knowledge in appropriate manner and in confidentiality (as appropriate).
- Maintain a fair and honoured and structured relationship – don’t become too involved in the person’s life, outside of professional relationship (e.g., don’t act as a service provider when you’re supposed to be co-researchers).
- Be mindful of wording for any written materials. If there is any possibility of triggers always have information on the bottom of all documents for resources.

**Transparency and accountability in decision-making**

Patient engagement liaison (with help of trainee) will keep detailed meeting notes that they will circulate after every meeting

PWLEs’ input and how it influenced decisions made within the study will be tracked and shared. Information tracked will include:

What did the PWLEs do?

What decisions did they make and why?

How did this impact the research and what changed as a result of engagement?

Every effort will be made to jointly arrive at consensus in decisions at meetings (decisions everyone can live with, even if they don’t necessarily agree)

**Expected outcomes**

- Expected outcomes for the academic research team include:
  - Preparing a scoping review protocol that will be submitted to an academic journal for publication,
  - Conducting the scoping review
  - Writing up the results of the scoping review for publication in an academic journal
  - Other knowledge translation activities (TBD)
    - Infographic for researchers about how to effectively engage patient partners
    - Infographics for patient partners about how PWLE can be engaged in scoping reviews
  - Preparing and submitting a paper that details our approach to patient engagement in scoping reviews (will include a subset of the study team + an additional researcher and trainee as co-authors)
    - What can PWLE hope to contribute and take away: A roadmap of what people were hoping to contribute. Influencing the scoping reviews' design and conduct
  - Possibility of future related research that PWLEs may have the opportunity to engage in (if interested)
- Expected outcomes for PWLEs include:

1) **What would you like to bring to this opportunity (e.g., your communities, your volunteer work, your work experience, your lived experience – speaks to positionality)?**

*Overall – aspects of your whole selves:*

- Interest
- Passion for advocacy and healthcare reform
- Previous experience with patient engagement and knowledge syntheses
- Lived experience (personal and of their communities) as a patient/care partner (general)
- Lived experience (personal and of their communities) as a patient/care partner in receipt of pharmaceutical services – good and bad, including: pharmacists potential and actual role in providing care and their scope of practice, importance of getting to meet and establish a relationship with a pharmacist and importance of having a set pharmacist instead of just rotating ones. Medication is a complicated and big thing for a lot of people. Connection is so important
- Work experience
- Education
- Volunteer experience (with health condition-related organizations)

*How do we know we’ve achieved this:* regular check-ins – asking PWLEs do you feel like you’ve had the opportunity to contribute your desired aspects of yourselves to our meetings?

2) **What do you hope to take out of this opportunity?**

- Working with new people
- Experience working on a scoping review
- Develop new skills and competencies
- Experience with patient engagement in research,
- Learn more about the world of research and medicine
- Collaborate with industry professionals
- Meet trained pharmacists/ researchers with a passion for improving pharmacy practice
- A step towards further enhancing and pushing forward towards more patient engagement in all aspects of healthcare
- Contribute meaningfully to the pharmacy industry

*How do we know we’ve achieved this:* Some of these outcomes will innately occur. For example, skill development, opportunities to review literature and contribute to making sense of findings, planned impacts on engagement in all aspects of health care, making a meaningful contribution to the pharmacy industry, and meeting other members of our research team.

3) **What does success look like for you (with this opportunity)?**

- working well together,
- bringing fresh perspective to the opportunity,
- evidence of meaningful contributions to team effort to develop creative innovation,
- accomplishing what we set out to do,
- personal fulfilment,
- positive impact on others

4) **How should we evaluate our engagement process (e.g., how does our work shape future engagement of patients in scoping review)?**

- Completing an anonymous evaluation form at the mid-point and end of the study. The form will assess the PWLEs’ engagement experiences, what is going well, and what could be going better in terms of:

A. Engagement processes:
   - Our working environment – whether it created the space for PWLEs to contribute and be heard
   - Ability to reach consensus
   - Whether PWLEs had a meaningful impact on the review (keep detailed records to observe changes made due to the engagement of PWLEs)
B. Review outputs:
   - How did engaging PWLEs shape the resultant research directions and outputs (e.g., resulted in more relevant research questions, improved methodological approaches, or increased dissemination and utilization of findings).
   - More traditional metrics of impact (e.g., citations of the review in subsequent research)
   - Influence on future research directions/processes (e.g., subsequent projects of this group and others)
   - Influence on changes in policies, guidelines or practices

**Our working environment**

The Canadian Institutes of Health Research’s Patient Engagement Framework outlines four guiding principles that should underlie patient engagement initiatives. We provide a brief overview of these guiding principles and how we will work together to co-create an environment that embodies these principles below:

A) **Mutual respect** - an environment in which PWLEs and other researchers acknowledge and value each other’s expertise and experiential knowledge.
   - Getting to know each other as individuals: Having an introductory meeting devoted to the patient engagement working group getting to know each other.
   - This could also be developed during introductions by having people share who they are as individuals and what they bring to the project (within and outside of their roles as patient, researcher, clinician, etc.).
   - This may be achieved through co-creating a Terms of Reference that identifies what people would like to bring to the project, establishes roles and responsibilities, and how the entire research team will work together.
   - Formally stating the value of lived experience and that it is a form of knowledge.
B) **Inclusiveness –** research that integrates a diversity of perspectives and reflects PWLE contributions.
   - This can be developed through maintaining a system of transparency by, for example, documenting decisions and next steps and why they did or did not happen.
   - Researchers asking and creating opportunities for people to be open and free to share their lived/living experiences (to the extent they feel comfortable), share what supports they need to feel welcome and included, and what they need in order to be able to contribute to the extent that they’d like to in the study.
   - Inclusion of a land acknowledgement in our presentations
   - We did not do this in our specific study, but for future recruitment. opportunities – making a clear statement in the initial recruitment posting that everyone is welcome. We could not reach consensus on whether an example of what is meant by the term everyone should be provided (as through these examples we are creating excluding groups when our intention is to be inclusive) in the posting, but a list that a patient partner shared from previous work included Indigenous peoples, BIPOC Canadians, newcomers to Canada, refugee status, 2SLGBTQ+, gender diverse individuals, people residing in rural communities, low income, neurodivergence, visible or invisible disability, other minority groups).
C) **Co-building** – an environment in which PWLEs and other researchers work together to identify problems/gaps, set priorities, and develop/implement solutions.
   - We are working towards this by beginning work on this project together in its early stages and planning to work together to its completion.
   - Shared-decision making: actively discussing and working through all aspects of the project in partnership with each other. Decisions are arrived at through conversation and consensus (i.e., decisions that not everyone may necessarily agree with, but can all live with).
D) **Supports** – an environment that supports PWLEs in contributing fully to discussions and decisions through:
i) **Creation of a “safe space**”
   - Respect for different opinions, backgrounds, and experiences.
   - Co-creating a Terms of Reference that makes it clear how we communicate
ii) **Educational supports**
    - We will work together to create a common language for the project and understanding of the tasks and components involved (through, for example, formally discussing what a scoping review is and what it involves when developing the protocol).
    - Creating a shared understanding of what a scoping review entails and potential ways to meaningfully contribute to it through sharing key literature and a webinar that provided an overview of patient engagement in scoping reviews
    - Offer opportunities for continuous learning beyond initial training, such as webinars, workshops, and/or seminars on emerging topics relevant to the scoping review and/or broader healthcare research. These opportunities are put forth by all members of our PWLE working group.
iii) **Financial (and non-financial) supports**

- We are working together to co-develop our hourly remuneration policy, including compensating PWLEs for prep time and are figuring out potential and creative ways to reimburse them for out-of-pocket expenses related to printing, etc.
- Making sure that all PWLEs are and feel recognized for their contributions to the scoping review. This could include public acknowledgment in presentations, publications, social media and personal acknowledgement of the value of the PWLE’s time and contributions to the study. It also includes co-authorship, certificates, plaques, considerations to PWLE’s CV building.
