# Supplementary material for: Facilitators and barriers to engaging patient partners in knowledge syntheses: A stage-based approach
Source: Res Synth Methods. 2026 Jun 15;17(6):1060–80. doi: 10.1017/rsm.2026.10099 (PMC13732379; doi:10.1017/rsm.2026.10099)
Supplement: Supplementary file 1 [file S1759287926100994sup001.zip › S1759287926100994sup001/Supplemental File 2 Recruitment Letter - new.docx]

**Recruitment Letter**

**Study background**

The importance of patient engagement in health care and research is becoming increasingly recognized, including its potential to improve care outcomes and research quality. In the field of pharmacy, patient engagement has become increasingly relevant with the evolution of the pharmacist’s role in patient-centered care, medication management, and disease prevention. Yet, despite the emphasis on patient-centered approaches in pharmacy, patient engagement is still uncommon in pharmacy practice research (defined as research about pharmacy practice or pharmacist-led patient care, where the population is pharmacists in a practice setting or the intervention and/or control is being delivered by a pharmacist in a practice setting). This includes, but is not limited to, clinical and outcome research, health services research, and comparative effectiveness research. Therefore, to help guide the growth of patient engagement in pharmacy practice research, we have set out to map the current state of patient engagement in pharmacy practice research.

**Methods – what will we “do” in this study?**

A scoping review is a type of study that aims to identify and summarize existing information (like peer reviewed journal articles) about a topic. We plan to develop a protocol for and carry out a scoping review of patient engagement in pharmacy practice research and publish peer reviewed journal articles about this work.

**Who are we looking to engage with?**

We are looking for approximately 2-4 patient partners to engage with us on this project. Since this work can be done virtually (via online meetings and independent computer-based work) patient partners can be located anywhere in Canada. However, they will need (a) reliable access to a computer or smart device and the internet, to attend team meetings and review study documents. Ideally, patient partners will also: (b) have previous experience engaging in pharmacy research, which they can apply to help steer our project, and (c) be able to meet virtually during regular working hours.

**Patient partners’ potential roles in this project**

**Scoping review protocol:** We have already begun developing the protocol for the scoping review, so patient partners’ preferred roles here would be to provide feedback and unique ideas to help us revise and optimize what we’ve developed thus far. If you’re familiar with the IAP2 Spectrum of Engagement (https://iap2canada.ca/Resources/Documents/0702-Foundations-Spectrum-MW-rev2%20(1).pdf) patient partners’ engagement will likely be at the level of “involve” given that we have already begun working on the protocol.

**Scoping review:** Ideally the same patient partners that engage on the protocol will stay on to “collaborate” (https://iap2canada.ca/Resources/Documents/0702-Foundations-Spectrum-MW-rev2%20(1).pdf) as equal members of our research team on the conduct of the actual scoping review. This means that ideally we want patient partners to have an active and “hands-on” role in the research activities that we undertake throughout the scoping review study. This includes engaging in regular meetings aimed at discussing scoping reviews progress, helping make sense of and guiding the writing up of the study findings, and could also include helping screen the articles for inclusion (if of interest to patient partners).

**Supports**

This project’s lead investigator has 5+ years of experience engaging patient partners in research, including on scoping reviews, which they will apply to ensuring the integrity of the engagement process. A study team member will act as an engagement liaison throughout the duration of your engagement. It will be their job to ensure that you are supported in engaging to your desired potential throughout the study. To ensure that everyone is on the “same page” about roles and responsibilities, at the start of the study, a team member will meet with patient partners to co-develop a terms of reference that outlines how we plan to work together on the study. Topics covered will include what the study is about; roles and responsibilities of study team members; what the terms “safety” and “meaningful contributions” mean/feel/look like to patient-researchers in the context of the study; the educational, practical, and other supports that patient partners need to collaborate on the study; and a workplan that includes major study milestones. The terms of reference is a “living document” that we will re-visit and revise as necessary throughout the duration of the study.

**Compensation**

We would like to compensate patients partners $25/hour for time spent on the project, including time spent preparing for meetings. Maximum allowable billable times for tasks (e.g., meeting prep, document review) will be mutually negotiated and agreed upon between patient partners and the engagement liaison to ensure transparency and project sustainability. We are also committed to exploring alternate forms of compensation for patient partners who are interested in this opportunity but may not be able to accept certain types of compensation. Patient partners will also be offered additional compensation through study authorship as long as they satisfy standard requirements for study authorship.

**Study timeline and estimate of time commitment**

As we have already begun working on our scoping review protocol, we would like to begin engaging patient partners as soon as possible in this project. We anticipate that the majority of the work on the scoping review will be carried out in the next six months (April to September), with more sporadic work involved as the scoping review goes through the peer review process (associated with submitting an article for publication in a research journal). We estimate that the time commitment will be around 8-12 hours each month, including prepping for and engaging in team meetings and carrying out project activities that patient partners are interested in contributing to.
