# Supplementary material for: Facilitators and barriers to engaging patient partners in knowledge syntheses: A stage-based approach
Source: Res Synth Methods. 2026 Jun 15;17(6):1060–80. doi: 10.1017/rsm.2026.10099 (PMC13732379; doi:10.1017/rsm.2026.10099)
Supplement: Supplementary file 1 [file S1759287926100994sup001.zip › S1759287926100994sup001/Supplemental File 3 Tech and accessibility needs survey - new.docx]

Technology and other accessibility needs survey

**Questions? Anything you’d like to discuss “in-person? Contact:**

[Redacted PWLE Liaison name and email] to set up a virtual or phone meeting

1.	How do you feel about participating in our meetings online?

2.	We want to ensure that you are able to fully participate:

a. Do you have a computer or mobile device (e.g., tablet, iPad, smartphone) with the internet?
b. Does it have a camera and audio?
c. Are you able to open and provide written feedback on documents sent as Word files or PDFs (no problem if the answer is no to either – we’ll figure out an alternative together)?

3.	Do you feel comfortable using Zoom? We can send you written instructions related to key Zoom features and/or set up a Zoom tutorial if needed,

4.	Are there any ability or health restrictions we need to be aware of to ensure we are meeting your needs?

5.	Has your availability changed?

6.	Do you have space in your residence (or anywhere else) where you feel comfortable sharing your thoughts and ideas freely?

7.	How do you prefer to provide feedback on study documents (e.g., I’m ok providing written feedback, I prefer to share my feedback verbally, etc.)

8.	Do you have any suggestions for ground rules/principals of working together when meeting online?

9.	Do you have any questions for me?

10.	Is there anything else you would like to talk to me about?
