# Supplementary material for: Facilitators and barriers to engaging patient partners in knowledge syntheses: A stage-based approach
Source: Res Synth Methods. 2026 Jun 15;17(6):1060–80. doi: 10.1017/rsm.2026.10099 (PMC13732379; doi:10.1017/rsm.2026.10099)
Supplement: Supplementary file 1 [file S1759287926100994sup001.zip › S1759287926100994sup001/Supplemental File 4 Training Resources - new.docx]

**Training Resource List**

1. Webinar: [**Bringing Patient Engagement in Health Research and Knowledge Translation Together**](https://www.youtube.com/watch?v=dgstBAdeHhY)
2. [**Unity Health Toronto – Patient & Community Engagement in Research Resource Guide**](https://research.unityhealth.to/resources/pcer/resource-guide)

This comprehensive guide is designed to assist researchers in effectively involving patients and community members in the design and implementation of research studies. Developed by Unity Health Toronto, the guide is structured into three progressive levels

- **Level 1: Learn** – Introduces the fundamentals of patient and community engagement in research.
- **Level 2: Apply** – Provides best practices for equitably engaging patients and community partners in research activities.
- **Level 3: Transform** – Focuses on advancing engagement practices through an equity, diversity, and inclusion lens.

Each level includes objectives, recommended resources, additional materials, and tracking forms to support self-directed learning. The guide emphasizes the value of incorporating lived experiences into research to enhance healthcare outcomes.
